# Assessing Metal Ion Assignment Accuracy in Protein Data Bank Models via Elemental Spectroscopy

**DOI:** 10.1021/acs.jcim.6c00352

**Published:** 2026-06-15

**Authors:** Edward H. Snell, Geoffrey W. Grime, Samuel M. Webb, Catia Costa, M. Elizabeth Snell, John F. Hunt, Liang Tong, Gaetano T. Montelione, Rachel Zigweid, Bart L. Staker, Peter J. Myler, Elspeth F. Garman

**Affiliations:** † The Department of Materials Design and Innovation, SUNY University at Buffalo, 120 Bonner Hall, Buffalo, New York 14260, United States; ‡ The University at Buffalo Hauptman-Woodward Institute, 700 Ellicott St., Buffalo, New York 14203, United States; § University of Surrey Ion Beam Centre, Guildford, Surrey GU2 7XH, U.K.; ∥ Stanford Synchrotron Radiation Lightsource, 2575 Sand Hill Road, MS69, Menlo Park, California 94025, United States; ⊥ Department of Biological Sciences, 5798 Columbia University, New York, New York 10027, United States; # Department of Chemistry and Chemical Biology, Center for Biotechnology and Interdisciplinary Sciences, 8024 Rensselaer Polytechnic Institute, Troy, New York 12180, United States; ∇ Seattle Structural Genomics Center for Infectious Diseases, Seattle, Washington 98109-5219, United States; ○ Center for Global Infectious Disease Research, Seattle Children’s Research Institute, Seattle, Washington 98101, United States; ◆ Department of Global Health, University of Washington, Seattle, Washington 98195, United States; ¶ Department of Biochemistry, Dorothy Crowfoot Hodgkin Building, University of Oxford, South Parks Road, Oxford OX1 3QU, U.K.

## Abstract

Accurate representation of metal ions in macromolecular structures is critical for chemical interpretation, computational modeling, and machine-learning methods that rely on Protein Data Bank (PDB) entries. However, the elemental identity of metals modeled in crystallographic structures is often inferred indirectly and rarely validated experimentally. Here, we combine Particle Induced X-ray Emission (PIXE) and X-ray Fluorescence Spectroscopy (XRFS) to determine the elemental composition of protein samples used to generate 70 deposited metalloprotein crystal structures. By analyzing the original protein material employed for crystallization, but before the addition of crystallization buffer solutions, we assess whether the modeled metal ions in deposited structures are consistent with experimentally detectable elemental content. We find that in a majority of cases, the metals modeled in the corresponding PDB entries are inconsistent with the metals present in the protein samples before crystallization, or that additional metals are present but not represented in the structural models. Spectroscopic results were integrated with automated crystallographic validation metrics, including real-space Z-difference (RSZD) analysis and systematic rerefinement, to evaluate atomic-number mismatch at metal sites. PIXE and XRFS show strong agreement for dominant elemental signals and provide complementary, scalable approaches for identifying suspect metal assignments. This work does not address physiological or functional metalation but instead highlights a widespread data integrity issue in deposited macromolecular structures, PDB-wide. These results establish an experimentally corroborated link between elemental identity and crystallographic validation metrics, enabling the large-scale detection of chemically inconsistent annotations in structural databases used for computational modeling and machine learning.

## Graphical abstract

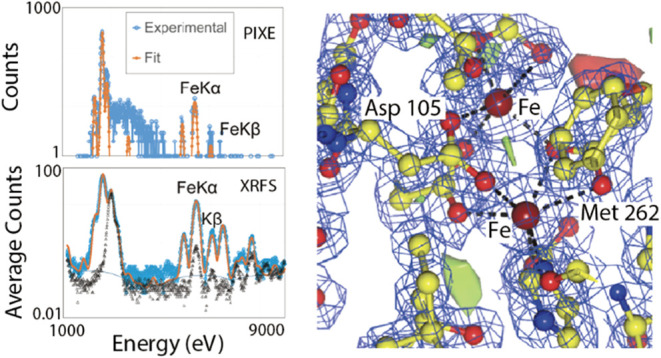

## 1 Introduction

Metals play important structural and catalytic roles in biological mechanisms.^1–3^ It has been estimated that 25–50% of the proteome has metal cofactors.^1–3^ Metalloproteins are significant in the progression of diseases, providing an attractive target for therapeutic intervention.^4^ Correct metalation is crucial for metalloprotein enzymatic activity and/or structure.^5^ However, proteins can be flexible and metalation can occur through tightly regulated networks of transporters, chaperones, and sensors that enforce metal homeostasis. When these systems fail, unwanted mis- or trans-metalation can occur, with profound biochemical and physiological consequences.

The general binding hierarchy of divalent metals follows the Irving-Williams series, Mg^2+^ < Mn^2+^ < Fe^2+^ < Co^2+^ < Ni^2+^ < Cu^2+^ (Cu^+^) > Zn^2+^ from weakest to tightest.^6^ For example, calculations^7^ show that all mononuclear Mg^2+^ sites in proteins bind Zn^2+^ orders of magnitude more tightly than Mg^2+^. However, selectivity is not dictated by thermodynamics alone; intrinsic preference can be governed by geometry and coordination number.^8^ Cofactor assembly defects can trigger iron mis-regulation and secondary metal stress that can cascade into mis-metalation elsewhere,^9^ and sensors and transporters exert tight control over metalation.^10^ Studies on intracellular Cu tracking proteins show that biological buffers, chaperones, and poising the protein in the correct redox state suppress mis-metalation.^11^

When confronted with equimolar mixtures of metal atoms, metalloproteins often bind metals that do not match their functional requirements. For this reason, metals are heterogeneously dispersed in cells, with particular metals being enriched in some locations and scarcer in others.^12^ Correct metal homeostasis in biological organisms is the result of detailed metal trafficking^13^ and careful incorporation of specific metals into a protein.^14^ These observations are directly relevant to structural biology: crystallographic models may capture metals that bind opportunistically during overexpression, purification, or crystallization, and unambiguous assignments can be compounded by the inherent challenges of distinguishing chemically similar ions. Thus, accurate identification of metals in the Protein Data Bank requires not only careful crystallographic validation but also consideration of biochemical homeostasis and established metalation pathways. In many cases, the low sample quantities of available protein also confound the issue.

By detecting the unique X-ray energy emitted by each element, Particle Induced X-ray Emission (PIXE) can be used to unambiguously identify metals present in a protein.^15,16^ PIXE measurements on a set of 30 metalloproteins, using the same protein samples from which models had been generated and deposited in the Protein Data Bank (PDB), showed that 56% of models do not accurately reflect the metal actually present in the precrystallization protein sample.^17^ In that study, a clear example of misidentified metals was the case of a putative histidinol phosphatase, hisK, from *Listeria monocytogenes* which has a trinuclear metal site. Using a combination of PIXE and anomalous X-ray signals present in the diffraction data, not only could the metals be identified as Fe, Co, and Mn instead of the Fe and Zn in the original model (OM), but they could also be modeled in the correct positions in the protein. In the OM the metal sites also contained a water cluster, but once the metal identities were corrected, this cluster was revealed to be a sulfate ligand in each site.^17^

The correct metal is key to mechanism, e.g., the replacement of Mn with Fe in superoxide dismutase greatly reducing activity,^18^ the dependence of electron tunneling on metal selection,^19^ and both tolerated and dysfunctional substitutions in cytochrome c.^20^

An incorrectly modeled metal can result in misinterpretation of biological mechanisms. For example, a metalloprotein from *Mycobacterium tuberculosis* was originally identified as a Ferric Uptake Regulator (Fur) by sequence homology.^21^ Members of the FUR family are central metal-dependent transcriptional regulator proteins in many bacteria. Iron ions were initially placed in the metal binding domains in the electron density for the *M. tuberculosis* putative FurB; PIXE data on the sample revealed that the protein actually bound three zinc atoms per protein, but no iron.^22^ The combination of the structural data, the PIXE metal identification and quantification, and the biochemical data, required a reinterpretation of the mechanism of action. It is now well-established that the FUR family contains proteins that sense zinc (Zur), manganese (Mur), and nickel (Nur), changing hypotheses about mechanistic details.^23^ Another example where PIXE analysis changed a structural interpretation was for a small G protein RhoAGDP/RhoGAP metal-nucleotide complex, where an alleged aluminum fluoride in the active site was actually magnesium fluoride.^24^ The transfer of phosphate groups is a key means of storing and transferring chemical energy as well as being mechanistically important for cellular signaling and regulation. PIXE analysis of this transition state analog was critical for mechanistic understanding but also in identifying magnesium fluoride as a reagent of choice for studying these phosphoryl transfer reactions.

Here we build on our initial high-throughput PIXE work by analyzing another 70 metalloprotein samples: 28 from the North East Structural Genomics Consortium (NESG), and 42 from the Seattle Structural Genomics Center for Infectious Diseases (SSGCID).^25^ The NESG was a large, multi-institutional research initiative funded by the National Institutes of Health (NIH) as part of the Protein Structure Initiative (PSI) and prioritized proteins with unknown structures, especially those from human, pathogens’, and model organisms’ genomes. The SSGCID is a consortium established to apply structural genomics approaches to potential drug targets from the National Institute of Allergy and Infectious Diseases (NIAID) priority organisms for biodefense, and emerging and re-emerging diseases.

We complement the PIXE measurements with X-ray fluorescence spectroscopy (XRFS), a more accessible technique due to the number of suitable synchrotron beamlines. By analyzing exactly the same protein samples that were used for PDB model deposition, we directly test the hypothesis that metals are often promiscuous, adventitiously incorporated, and misidentified when not explicitly validated. Our results, exploiting XRFS, confirm our initial experimental finding^17^ from structural genomics center efforts, that a significant proportion of metalloproteins deposited in the PDB by both structural genomics and nonstructural-genomics efforts may have incorrectly modeled metals either due to misidentification, or by incorporation from the crystallization conditions.

In this study, we identify the elemental content of the original precrystallization protein sample used to generate a deposited crystallographic model and demonstrate how frequently those models misrepresent that elemental identity. Our conclusions are therefore restricted to the elemental identity within the measured samples. The metal content in heterologously expressed proteins may not reflect the physiological metalation state *in vivo*. Inference of the native, functional metalation state would require complementary *in vivo*/biochemical validation of the type routinely performed by many laboratories specializing in metalloprotein studies. Extreme care is required to accurately characterize the functional metal. Unfortunately, a significant number of those who determine metalloprotein structures are not metalloprotein specialists and may not have particular expertise in metal identification. The problem is significant. From 2018 to the end of 2024, there were 9,338,167,995 models downloaded from the PDB, and conservatively, ∼3000 million of those were metalloproteins.^26^ The PDB curators estimate that over 99% of downloads are not by structural biologists and the statistical data show that only ∼4% of any structural models are accompanied in the download by their experimental data. Many researchers who use these models, both inside and outside the field of structural biology, may be unaware of the possibility of incorrect metal identities in the models, or even how to detect them. The growth of artificial intelligence and the use of these flawed data as a training set only confounds the problem, yielding erroneous predictions and output.

## 2 Experimental Methods

### 2.1 Computational Analysis of Experimental Data from Metalloproteins in the PDB

To identify suitable candidates for the extended study, we first conducted a computational analysis of metalloprotein models in the PDB. Two sets of metalloprotein model PDB IDs were selected, the first chosen at random from samples submitted by the NESG to the Crystallization Center at the Hauptman-Woodward Medical Research Institute (HWI) and a second more targeted set from models deposited by the SSGCID. Analysis of the PDB models and deposited experimental data was performed with a script named Alchemy that automates several analysis steps that would otherwise have to be carried out separately. This script downloads the molecular model and experimental structure factors from PDB-REDO.^27,28^ It then calls a set of CCP4 ^29^ programs to calculate the associated *2Fo-Fc* and *Fo-Fc* difference electron density maps for each structure. The program EDSTATS^30^ is run to calculate a difference density Δρ/σ­(Δρ), or *Z* score, for each residue and ligand (*RSZD*) as a quality indicator. This parameter has both sign and magnitude. A negative *RSZD* implies that the model has more electrons than the experimental data indicate (too high an atomic number), while a positive *RSZD* implies that too few electrons are modeled compared to the experimental data (too low an atomic number or less than 100% occupancy). The real space *R* value (*RSR*), where *RSR* = ∑|ρ_obs_ – ρ_calc_|/∑|ρ_obs_ + ρ_calc_| and that has a range of 0 (good) to 1 (poor), is also calculated. These quality indicators are computed for all residues and metal atoms (note that when we refer to the *RSZD*, we refer to that of the largest *RSZD* associated with a metal in the model). The script then calls CCP4 mg^31^ to produce images of the metal environment and the two electron density difference maps, and the numerical results are tabulated. The model and structure factors are taken from PDB-REDO using the most current version (8.13) to try to minimize the subjective human element and ensure that any algorithmic developments are taken into account since the OM was first determined. The NESG set was used to test the predictive capability of the Alchemy method to identify suspect metal sites in deposited models, and the SSGCID deposited models were curated to identify targets for PIXE and XRFS measurements. Both sets of proteins included those with suspect and good *RSZD* scores for the metals in the OMs.

### 2.2 Sample Selection

The selected samples and RSZDs are detailed in Tables S1 and S2 of the Supporting Information with the protein names and PDB IDs listed in Tables 1 and 2. The samples were taken from the very same protein preparations used to grow the crystals that generated the original diffraction data and the deposited models in the PDB. The proteins were not produced afresh, repurified, or altered in any way except for having been in long-term storage at −80 °C.

**Table 1:** Results from the PIXE Data for the NESG Samples

#	PDB ID	description	metal in PDB	RSZD	PIXE metal/signal^a^	PIXE Ratios^b^	calculated metal atoms per protein molecule based on Se ratio^c^	crystallization cond. metal/s
**NESG class I proteins: PDB original model inconsistent with PIXE data**								
1	3vb9	unknown function	Mg	16.2 (Mg)	K, Se	0.66/0.34	8 K, 1 Ca, 1 Fe	Mg
2	4py9	exopolyphosphatase-related protein	Na	12.4(Na)	Se (Fe,Mn)	1.0	1 K, 1 Ca, 1 Mn, 1 Fe	K, Na
4	3mel	Thiamin pyrophosphokinase family protein	Mg	10.8 (Mg)	Fe (K)	1.0	1 K, 1 Fe	Na, Mg
5	2f6k	Amidohydrorolase II	Mn	9.4 (Mn)	(K, Fe)	N/A	N/A	Mg
7	2gzx	TatD deoxyribonuclease	Ni	7.4 (Ni)	K, Fe, Se	0.86/0.07/0.07	23 K, 2 Fe	Mg
9	3bb6	P64488 protein	Zn	6.9 (Zn)	Se, K (Fe)	0.72/0.28	none	Na
10	3guw	TatD-like Protein	Zn	6.7 (Zn)	none	N/A	N/A	Mg
11	2h6l	unknown function	Zn	–6.4 (Zn)	K, Ca, Se (Fe, Co)	0.86/0.07/0.07	11 K, 1 Ca, 1 Zn	Na
13	2bdr	Putative Ureidoglycolate hydrolase	Na	8.6 (Na)	Se, K (Mn, Fe)	0.60/0.40	1 K	Na
14	2gwg	4-Oxalomesaconate Hydratase	Zn	–5.1 (Zn)	Se (K)	1.0	3 K, 1 Ca, 1 Co	Na, Li
17	2gsl	unknown function	Mg	3.9 (Mg)	Ca, K	0.88/0.12	N/A	none
20	4jc0 ^e^	*Thermotoga maritima* holo RimO	Fe, Na	2.65	K, Ca (Se)	0.77/0.23	31 K, 10 Ca	Na, Fe
21	2gta	putative pyrophosphatase	Na	2.2 (Na)	Se, Ca, K (Fe)	0.66/0.20/0.14	none	Na
22	2rjb	unknown function	Zn	2.0 (Zn)	K, Fe (Ca)	0.81/0.19	11 K, 5 Ca, 3 Fe, 1 Ni, 1 Co	none
23	4fj4	Unknown function	Na	–2.0 (Na)	K, Ca	0.56/0.46	9 K, 7 Ca,	Na
24	3c5m	oligogalacturonate lyase (VPA0088)	Mn	1.8 (Mn)	Se (Ni)	1.0	2 Ni, 1 Ca	none
26	3cew	cupin protein (BF4112)	Zn	–1.2 (Zn)	Ca, Fe, Se	0.52/0.33/0.15	4 Ca, 1 Fe	Na, Mg
28	1ydn	HMG-CoA Lyase	Ca	N/A^d^	none	N/A	N/A	Ca
								
**NESG class II proteins: Extra metals indicated by PIXE data**								
6	3sm3	SAM-dependent methyltransferases Q8PUK2_METMA	Ca	8.5 (Ca)	Ca, K	0.87/0.13	31 Ca, 5 K	Ca
8	2hf1	putative Tetraacyldisaccharide-1-P 4-kinase	Zn	7.0 (Zn)	Ca, Se, Zn, Co	0.49/0.22/0.18/0.11	1 Ca, 1 Zn	Na
12	2h28	unknown function	Mg	–5.9 (Mg)	Se, Fe (Ca)	0.78/0.22	none	Mg
15	4h9d	Mn-dependent Gme HNH nicking endonuclease	Zn, Mg	4.4 (Mg)	Co, K, Zn (Se)	0.40/0.30/0.26	2 Zn, 1 K, 1 Co	Na, Mg
19	2o18	Thiamine biosynthesis lipoprotein apbE	Ca	2.5 (Ca)	Se, K (Ca)	0.63/0.37	2 K, 1 Ca	Li
25	2r7d	ribonuclease II family protein	Mg	1.6 (Mg)	K, Ca, Se (Mn)	0.42/0.39/0.19	8 K, 8 Ca, 1 Mn, 1 Fe	Mg
27	2nvv	Putative Acetyl-CoA hydrolase/transferase	Fe	1.1	Se, Ca, K (Fe)	0.46/0.29/0.25	3 Ca, 3 K	Ca
								
**NSEG class III: PIXE in agreement with the original model**								
3	3goc	Endonuclease V (SAV1684)	Mg	–12.2 (Mg)	none	N/A	N/A	Na, Mg
18	3c37	putative Zn-dependent peptidase Q74D82	Zn	5.7 (Zn)	Se (Zn, K)	1.0	3 Zn, 1 K	Na
16	3kb2	unknown function	Mg	4.1 (Mg)	(K)	N/A	N/A	Na, Mg

a Metals detected by PIXE are noted if the signal is >3 × LOD; if >2 × LOD, then they are noted in parentheses.

b The ratio is given by the metal signal if it is >3 × LOD, divided by the total sum of all signals that are >3 × LOD for that sample. This is a ratio of the signal recorded and is not normalized to the molecular weight.

c The number of Se atoms for most of the samples is known and this is used to calculate the nearest integer number of other metal atoms per protein molecule, irrespective of the LOD. For those with no Se present, the ratios of the metal signals are provided.

d No experimental data were available in the PDB for these models.

e The sample contained a ligand with an iron sulfur cluster, not a metal ion bound to the protein.

**Table 2:** Results from PIXE and XRFS Data for the SSGCID Samples

#	PDB ID	description	Metal in PDB	RSZD	PIXE metal/s	PIXE Ratios^a^	XRFS metal/s	XRFS Ratios^b^	Crystallization cond. metal/s
SSGCID class I: PDB original model inconsistent with PIXE data and/or XRFS data									
4	5b8f	2-C-methyl-D-erythritol 2,4-cyclodiphosphate synthase	Mg	17.6 (Mg)	Fe, Mn, Ni, Zn	0.36/0.25/0.21/0.18	Zn, Fe, Cu, Ni, Mn	0.59/0.17/0.15/0.06/0.03	Na
6	5uf2	Ribose-5-phosphate isomerase A	Na	16.7 (Na)	Ni, Zn	0.72/0.28	Zn, Ni	0.55/0.45	Na
8	4pcl	O-methyltransferase family protein	Mn	–12.5 (Mn)	Ni (Fe) (Mn was present at >1.5 × LOD)	1.00	Zn, Cu, and Ni are present at (>1.5 × LOD)	none	Na
9	4hdt	3-hydroxyisobutyryl-CoA hydrolase	Zn	–12.4 (Zn)	Ca	1.00	Ca (Se)	1.00	Zn, Na
10	4u7x	Ribokinase:Carbohydrate kinase, PfkB	Na	12,1 (Na)	Ni, Zn (Cu, Se)	0.94/0.06	Ni	1.00	Na
13	4y0e	Putative dioxygenase	Fe	–11.3 (Fe)	Ni (Zn)	1.00	Ni, Zn (Ca)	0.53/0.47	Mg, Ca, Na
14	6ao1	β-lactamase domain protein	Ca	10.8 (Ca)	Fe, Zn, Mn, Ni, Cu, Se	0.50/0.26/0.15/0.07/0.01/0.01	Zn, Fe, Ni, Mn, Se (Co) (Cu is present at >1.5 × LOD)	0.45/0.44/0.04/0.04/0.03	Ca, Na
19	5unb	Putative deoxyribonuclease-2	Cu	–8.2 (Cu)	Ca, Ni	0.65/0.35	Ca and Ni were the maximum signals but at (<1 × LOD)	none	Cu
21	4o5f	Type III pantothenate kinase	K	–5.8 (K)	Ni (Se)	1.0	Ni (Se)	1.00	K, Mg, Na
22	3mx6	Methionine aminopeptidase	Mn, Na	–5.7 (Mn)	Fe (Mn)	1.0	Fe, Ni, Mn (Zn is present at >1.5 × LOD)	0.79/0.12/0.09	Na
27	3oce	Fumarate lyase:Delta Crystallin	Co	–4.0 (Co)	Zn, Ni (Ca)	0.66/0.34	Zn	1.00	Co, Na
28	3u0a	Acyl-CoA thioesterase II TesB2	Na	3.8 (Na)	none	none	none	none	Na
31	6n1f	Oxidoreductase, 2OG-Fe(II) oxygenase family	Fe	–2.4 (Fe)	(Ni is present at >1.5 × LOD)	none	Ni, Zn	0.89/0.11	Na
33	3u0f	β-ketoacyl synthase	Na	–2.2 (Na)	Ca, K (Ni)	0.41/0.59	(Ni is present at >1.2 × LOD)	none	Na
34	3r1i	Short-chain type dehydrogenase/reductase	Mg	2.1 (Mg)	Ca, Ni, Fe	0.92/0.05/0.02	Ni (Ca is present at >1.5 × LOD)	1.00	Mg, Na
38	3r4t	4-aminobutyrate aminotransferase GabT	Na	1.0 (Na)	Ca	1.00	none	none	Na
42	5i3e	Putative deoxyribonuclease-2	none	N/A	Ni, Ca (Cu)	0.68/0.32	Ni, Zn, Se	0.64/0.18/0.18	Na
SSGCID class II: Extra metals indicated by PIXE and/or XRFS data									
1	4o5o	3-hydroxyacyl-CoA dehydrogenase	Zn	–24.3 (Zn)	Ni, Zn	0.93/0.07	Ni (Zn)	1.00	Na
3	3uw2	Phosphoglucomutase/phosphomannomutase family protein	Zn	–18.3 (Zn)	Mn, Zn, Fe, Ni	0.45/0.20/0.18/0.17	Zn, Fe, Ni, Mn	0.41/0.24/0.18/0.17	Na
4	4wok	UDP-glucose 4-epimerase	Zn	–17.4 (Zn)	K, Zn Fe, Ca	0.70/0.13/0.10/0.07	K, Zn, Fe, Ca, Ni	0.78/0.15/0.05/0.02/0.01	Na
6	4dz4	Agmatinase	Mn	–13.7(Mn)	Fe, Mn, Ni, Zn (Ca)	0.47/0.47/0.05/0.01	Fe, Mn, Ni (Se and Zn are present at >1.5 × LOD)	0.78/0.19/0.03	Na
10	3r1j	α-ketoglutarate-dependent taurine dioxygenase	Fe	–11.9 (Fe)	Ca, Ni, Fe	0.92/0.05/0.02	Ni	1.00	Na
11	4wjb	putative amidohydrolase/peptidase	Zn	–11.6 (Zn)	Zn, Fe, Ni (Cu)	0.63/0.24/0.13	Zn, Fe, Ni, Se	0.79/0.10/0.08/0.04	Na
14	5umh	catechol 1,2-dioxygenase	Zn, Ca	–10.8 (Zn)	Fe, Zn, Ni (Ca)	0.67/0.24/0.09	Zn, Fe, Ni	0.51/0.40/0.08	Fe, Zn, Ca, Na
15	4f0l	Amidohydrolase from *Brucella melitensis*	Fe	–10.4 (Fe)	Zn, Fe, Ca, Mn (Ni)	0.47/0.34/0.10/0.09	Fe, Ni, Mn, Zn	0.60/0.20/0.11/0.10	Na
16	3s6l	Hep_Hag family	Zn	10.1 (Zn)	Ni, Zn, Cu	0.61/0.21/0.18	Ni	1.00	Na, Zn
17	5u8o	Zn-dependent hydrolase	Zn, Ca	–9.1 (Ca)	Fe, Mn, Zn, Ni (Se)	0.61/0.23/0.10/0.06	Fe, Zn, Mn, Se, Ni	0.61/0.21/0.08/0.06/0.05	Ca, Zn, Na
19	4di0	Rubrerythrin	Fe	–8.1(Fe)	Fe, Mn, Zn, Ca (Ni)	0.59/0.21/0.12/0.08	Fe, Zn, Mn, Ni, Ca, Co	0.61/0.24/0.09/0.03/0.01/0.01	Fe, Ne
20	5kob	Peptide deformylase	Fe	–6.0(Fe)	Fe	1.00	Zn, Fe, Ni	0.55/0.34/0.11	Mg, Na
22	6n7l	Alcohol dehydrogenase	Zn	–5.3 (Zn)	Zn, Ni, Fe	0.82/0.10/0.08	Zn, Ni	0.96/0.04	Na
23	6d6j	HIT family hydrolase	Zn	5.3 (Zn)	Zn, Ni, Fe, Co (Se)	0.93/0.04/0.01/0.01	Zn, Ni, Se	0.97/0.02/0.01	Na
24	3md7	β-lactamase-like	Mn, K, Na	5.1 (Mn)	Mn, Fe, Ni, Zn, Se, Ca (K)	0.65/0.14/0.09/0.08/0.02/0.02	Fe, Mn, Zn, Se, Ni (Ca)	0.43/0.25/0.17/0.09/0.05	K, Na
25	4j5i	α-ketoglutarate-dependent taurine dioxygenase	Fe, Mg	4.6 (Fe)	Ni, Fe (Zn)	0.87/0.13	Ni, Zn, Fe (Cu)	0.81/0.11/0.08	Mg, Li, Na
26	4yet	Superoxide dismutase	Fe	3.3 (Fe)	Mn, Fe, Ni (Ca)	0.47/0.46/0.08	Fe, Mn, Ni, Se, Zn (Co)	0.68/0.17/0.10/0.02/0.02	Fe, Na
29	5umf	Ribulose-phosphate 3-epimerase	Zn	–2.5 (Zn)	Zn, Fe (Ni)	0.65/0.35	(Zn, Ni)	none	Zn, Na
30	4gri	Glutamate--tRNA ligase	Zn	2.4 (Zn)	Zn, Ca, Ni	0.56/0.38/0.06 (Fe was present at >1.5 × LOD)	Zn (Ca) (Ni is present at >1.5 × LOD)	1.00	K, Na
32	3o0m	HIT family protein	Zn	–2.4 (Zn)	Zn, Ni	0.96/0.04	Zn, Cu (Ni is present at >1.5 × LOD)	0.98/0.02	Ca, Na
35	3omf	Putative histidine triad family protein	Zn	1.8 (Zn)	Zn, Fe, Ni, Ca	0.76/0.10/0.10/0.05	Zn, Fe, Se (Ni)	0.93/0.05/0.01	Na
36	3r6f	HIT family protein	Zn	1.6 (Zn)	Zn, Ca (Co)	0.88/0.12	Zn, Se (Co)	0.98/0.02	Zn, Na
37	6caz	Peptide deformylase	Ni	1.3 (Ni)	Zn, Ca (Ni)	0.65/0.35	Zn, Ni (Se)	0.93/0.07	Na
40	3oca	Peptide deformylase	Zn	0.8 (Zn)	Zn (Ca, Cu)	1.00	Zn (Ca, Se)	1.00	Zn, Na
39	5j46	Peptide deformylase 1	Zn	0.9 (Zn)	Fe (Zn)	1.00	(Zn)	none	Zn, Na

a As in Table 1, the ratio is given by the metal signal if it is >3 × LOD, divided by the total sum of all signals that are >3 × LOD for that sample.

b In the SSGCID case, Se-MET is present for most of the samples. The concentration of the metal atoms is directly measured and the ratio calculated for all those >3 × LOD or greater taking into account the molecular weight. Metals detected with PIXE and XRFS are noted if the signal is >3 × LOD; if >2 × LOD, they are noted in parentheses. Where a metal is seen by PIXE and it is also present in the XRFS spectra, it is noted with an indication of the LOD. The metals are listed in the order of concentration from high to low in each case. Most samples display a Ni signal present, presumably from the purification process. For protein #1, 4o5o, if the Ni signal were ignored as a common contaminant, the protein could be classified as class III. However, Ni explains the large difference electron density signal seen, and therefore justifies the classification.

The NESG proteins were originally produced with MJ9 minimal medium, enriched in metals and vitamins^32^ with most supplemented with selenomethionine (SeMet) for crystallographic phasing purposes as described elsewhere.^33,34^ All were in a buffer containing 5 mM dithiothreitol (DTT), 100 mM sodium chloride (NaCl), and 10 mM tris hydrochloride (Tris–HCl) pH 7.5, and in some cases included 0.02% sodium azide (NaN_3_). In the PIXE experiments the presence of sulfur in DTT means that the S signal could not be used for internal calibration but that the signal from Se could, as utilized in our original high-throughput PIXE study.^17^

The structural models of all the NESG samples were determined as part of the PSI, using the same protein sample as produced for crystallization screening which was carried out at the HWI Crystallization Center. All models (apart from one model determined some time ago) were deposited in the PDB together with their structure factors and with the crystallization conditions optimized from the original crystallization screening.

The SSGCID protein production pipeline is also well described elsewhere.^35^ Once expressed and purified, the individual proteins were typically concentrated into a final buffer containing chlorine, sodium and sulfur (25 mM 4-(2-hydroxyethyl)-1-piperazineethanesulfonic acid (HEPES)) pH 7.0, 500 mM NaCl, 5% glycerol, 2 mM Dithiothreitol (DTT), and 0.025% NaN_3_. The presence of HEPES (which also contains sulfur) in the samples and lack of Se mean that qualitative results can be acquired, but quantitative results cannot be reliably extracted from the PIXE measurements. The purification workflow for these samples includes nickel-based immobilized metal affinity chromatography (IMAC), a potential source of adventitious metal incorporation. In the SSGCID pipeline, proteins are deliberately re-exposed to Ni^2+^-charged resin in a second IMAC step following His-tag cleavage, whereas NESG samples were typically purified using a single affinity step.

No crystallization reagents, precipitants, salts, or additives were introduced to the samples analyzed by PIXE or XRFS; all measurements were performed on archived aliquots of the original purified protein preparation. We note that some metalloproteins can be sensitive to oxidation, and anaerobic handling is best practice for their study. For both the NESG and SSGCID samples, no anaerobic procedures were used in their preparation, the original X-ray crystallography studies, their storage, or in the spectroscopic studies described here.

### 2.3 Particle Induced X-ray Emission Studies

Samples for PIXE analysis were manually pipetted onto 4 μm thick polypropylene support film (TF-240, Fluxana, Germany) stretched and secured across five 8 mm × 8 mm square apertures (windows) in an aluminum alloy plate (cassettes). Up to three different proteins were placed onto each aperture. The droplets of each protein, each of between 0.1 and 0.2 μL, were arranged in unique geometrical patterns with at least three individual droplets for each protein, and the drops were allowed to dry out at room temperature. The patterns of dry drops provided a visual control to ensure accurate identification of each sample in both the PIXE and subsequent XRFS studies. Cross-talk between samples had previously been investigated by sampling an array of dried drops of different metal salts at 200 μm separation. Cross-talk between them was not present above the limits of detection (LODs) at the sample spacings used for the study here.

The PIXE analysis, from dried samples at ambient temperature, was carried out using the microbeamline on the 2MV Tandetron accelerator (HVEE Corporation, NL) installed in the Stephens Laboratory of the University of Surrey Ion Beam Centre.^36^ Monoenergetic 2.5 MeV protons (available energies 1.5 to 4 MeV) are focused to a diameter of <2 μm using a magnetic quadrupole triplet lens type OM-150 (Oxford Microbeams Ltd., UK).^37^ The beam is transported under high vacuum, and analysis takes place in an evacuated target chamber. The focused beam can be magnetically scanned over areas of up to 2 × 2 mm^2^. The beam induced X-rays are detected using a silicon drift detector (Rayspec, UK) with an active area of 80 mm^2^, active layer thickness of 0.5 mm and 140 eV resolution at an X-ray energy of 5.9 keV. This is mounted at a scattering angle of 135° to the beam direction in the horizontal plane and with a sample to detector distance which can be varied between 20 mm and 70 mm. Several measures were taken to reduce background signals and improve spectral quality. To stop recoiling ions entering the detector, it was fitted with a 130 μm thick beryllium absorber. Also, to reduce the intense chlorine signal by approximately 50×, an additional X-ray filter consisting of 100 μm Kapton foil (2 × 50 μm foils with a nominal total thickness of 100 μm) was used. This filter did not allow the measurement of X-rays from Na or Mg. Finally, a conical collimator was used to screen spurious signals from the rear of the target chamber from entering the detector. Elastically (Rutherford) backscattered (RBS) protons are detected simultaneously using a PIPS charged particle detector (Ametek Ortec, UK) with an active area of 150 mm^2^ mounted 52.5 mm from the sample and at a scattering angle of 160° in the vertical plane. X-ray photon and particle energies are detected as amplitude-modulated voltage pulses, digitized and recorded using the OMDAQ data acquisition system (Oxford Microbeams Ltd., UK). OMDAQ also controls the magnetic beam scanning and the positioning of the motorized sample stage, allowing the recorded data pulses to be coordinated with the beam position to create elemental maps.

The RBS proton spectrum is collected in order to allow the sample matrix composition and thickness to be well determined, enabling accurate correction of the PIXE data for sample matrix effects (X-ray absorption and incident particle energy loss within the sample).^6^

For the present study, three experimental PIXE runs were carried out: one on the NESG samples, and two on the SSGCID samples. We denote these runs as two to four with the number one being assigned to that described in our original high-throughput PIXE study.^17^ For the first drop of each protein studied, the spectra were monitored in real time, and the collection was terminated when sufficient counts were accumulated to give usable statistics in the peaks of interest. Collection from subsequent drops of the same sample were continued until the same beam charge had accrued. The beam charge required per analysis point was typically 500 nC or approximately 10 min collection time, depending on the beam current. Note that with the protective beryllium filter fitted on the X-ray detector that was used, the analysis was insensitive to Mg and lighter atomic number elements. The Se-Met incorporation in the NESG samples (run 2) allows the stoichiometry of metals to be calculated. No Se-Met was incorporated into the SSGCID samples (runs 3 and 4), and a simple semiquantitative ratio of the signal from different metals, excluding Se, was determined for both sets of samples. Since there was sulfur in the buffers in addition to the sulfur in the protein, the usual PIXE stoichiometric analysis could not be utilized. The experimental arrangement and methodology have been well described elsewhere.^15–17^

### 2.4 X-ray Fluorescence Spectroscopy (XRFS)

Because PIXE and XRFS are atomic analysis techniques insensitive to the state of the protein, the PIXE analysis cassettes could be used for the XFRS studies, despite the latter experiments being carried out some time later. The NESG set of proteins analyzed by PIXE were not available for the XRFS studies. However, the SSGCID cassettes were still accessible and were transported from the PIXE facility in the UK to the Stanford Synchrotron Radiation Lightsource (SSRL).

Data were acquired at ambient temperature on beamline 7–2 of SSRL from the original cassettes used in the PIXE studies, which had also been stored at ambient temperature. SSRL was operating at an electron energy of 3-GeV, with a targeted ring current of 450–500 mA, operating in top-up mode. Beamline 7–2 is equipped with a 20-pole 2-T wiggler, using a liquid N_2_-cooled Si(111) sagittal focusing double-crystal monochromator (operating in the ϕ = 0° orientation) and a Rh-coated vertically focusing flat bent mirror. A microfocused beam was achieved using an aperture downstream of the *I*_0_ ion chamber, followed by a microfocusing polycapillary to achieve a spot size at the sample of 50 μm in diameter. As for the PIXE experiments, cross talk was experimentally investigated by comparing the X-ray spectra from a dried drop and from the space between them. No signals from neighboring samples were detected at the spacings used.

The sample cassettes that had been used for the PIXE studies were mounted 45° to the incident 13.450 keV X-ray beam using double sided scotch tape on a frame, and the windows in the holder were raster scanned using Newport IMS Series stages (Irvine, CA) with a continuous horizontal motion summed over 50 μm intervals and successively stepped 50 μm vertically after each horizontal scan. The data acquisition rate was set at 50 ms per interval. A silicon drift Hitachi Vortex detector using six identical detection channels was positioned normal (i.e., at 90°) to the sample. The detector was fitted with a collimator to reduce the total counts from scattered X-rays in the sample area and the combined multichannel array spectrum for each interval was saved for off-line processing. The data are displayed as an image composed of individual ’pixels,’ each representing a 50 μm × 50 μm rastering interval in a two-dimensional (*x*–*y*) positional array. A full energy spectrum is accumulated for each pixel. The scan areas for each of the five cassette windows were manually defined as single imaging regions, with data collection for each window taking approximately 30 min. The experimental arrangement is shown in Figure 1.

**Figure 1:**
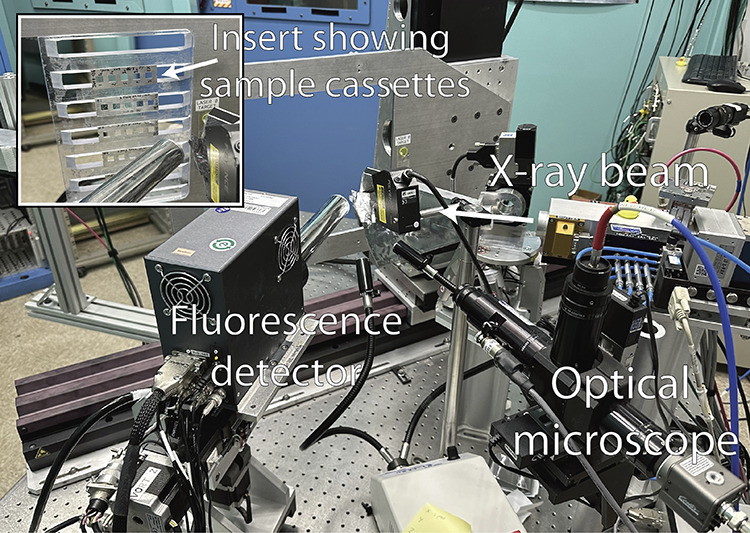
Experimental arrangement for the XRFS experiments on beamline 7–2 at SSRL. The sample mount is empty in the main image, but four sample cassettes are shown mounted in the top left insert of the figure.

Multichannel array data from the XRFS data were processed using the MicroAnalysis Toolkit SMAK.^38^ When each drop is selected manually, the PyMCA utiity^39^ incorporated into SMAK is used to fit the background, identify characteristic element peaks, and fit a calculated spectrum to the experimental one, taking into account the 4 μm thick polypropylene backing, the air path, and the detector material. The windows for each cassette were processed individually with drops of each sample manually selected and a spectrum obtained from averaging over every pixel making up the drop. For each element identified, the area under the fitted peak was integrated. The associated uncertainty is calculated based on the statistical noise in the spectrum and the quality of the model fit indicated by residuals between the data and fitted spectrum. All the drops for a single sample were summed into a weighted average, calculated based on the uncertainty. A blank area of the cassette window was defined, and a spectrum was obtained by summing the pixels in this area. This allowed the signal from the backing film without sample to be determined, along with an uncertainty in this signal. An LOD was determined by calculating the uncertainty in the mean, and a signal was considered significant when it was 3× this uncertainty or greater, above that of the area with no sample. The signal is proportional to the elemental concentrations and all were normalized to molar ratios. The ratio of the concentrations of the metals present above 3 × LOD within each sample was used due to differences and any uncertainty in the original sample protein concentration, so that comparison was made within the sample, and not between different samples.

Reference spectra were collected using thin-film calibration standards deposited on a 6.3-μm-thick mylar film and held on a frame in the sample holder position. Standards corresponded to the following quantities of the respective elements: K and Cl (KCl, 49.1 μg/cm^2^), Ca (CaF_2_, 56.8 μg/cm^2^), P (GaP, 47.0 μg/cm^2^), Fe (Fe, 56.0 μg/cm^2^), S and Cu (CuS, Cu = 39.4 μg/cm^2^, S = 11.8 μg/cm^2^), Mo (MoO 351.7 μg/cm^2^), Zn (ZnTe, 45.8 μg/cm^2^), Ni (55.4 μg/cm^2^), Co (45 μg/cm^2^) and Mn (47.1 μg/cm^2^) (Micromatter, CA, USA). These were used to determine the sensitivity of the technique.

### 2.5 Rerefinement of the Deposited Models

The number of proteins studied was large, so where there was a clear indication of a misidentified metal being present in the PDB atomic model, the metal was replaced with those detected in the PIXE and/or XRFS experiments, which were carried out on the sample before the crystallization components were added. PDB-REDO^27,28^ version 8.13 was then run with the deposited X-ray data but with the originally modeled metal replaced by one or more of the PIXE identified ones. A single run of PDB-REDO was conducted on the OM with the new metal information, and the maps examined. The *R* and *R*_free_ were compared to those calculated from the OM. In Tables S1 and S2, the *R* and *R*_free_ values from the PDB deposition are listed. Comparing these with the PDB-REDO values helps eliminate any human bias in the refinement process. If a metal that agreed more closely with the electron density was identified by the PIXE and XRFS data, this was substituted into the OM, and further rerefinement took place for a limited number of the proteins. For a small number of cases, although the metal identity was experimentally confirmed, the map showed negative electron difference density, indicating that the metal site was not fully occupied. REFMAC5 was used on its own to refine the occupancy of these sites, and that occupancy was then used in a subsequent PDB-REDO refinement. Without the availability of anomalous single crystal X-ray diffraction data, or complementary techniques, it was not possible to unambiguously resolve cases where two or more different metals may be partially occupying a metal site, since no information on atomic positions is obtained from either PIXE or XRFS data. In a number of cases, the CheckMyMetal server^40^ (CMMS) was used to evaluate geometrical features of the model, supplementing the analysis of experimental diffraction data and other measurements on the protein itself. The CMMS qualitatively evaluates the occupancy, the *B*-factor, the elemental composition of the coordination sphere, the summation of bond valence values, the summation of ligand vectors (which indicate when the coordination sphere is not symmetrical), the geometry, and vacancies in the coordination sphere etc. as dubious, borderline, or acceptable based on statistics of metals in the PDB,^3^ and proposes a list of alternate metals if applicable.

## 3 Results

Similar to our previously reported PIXE analysis of 30 metalloproteins,^17^ the results from all 70 proteins were arranged into distinct classes based on the PIXE results. These were: class I, where the OM and PIXE results were inconsistent (35 or 50%); class II, where the modeled metal was present (or there was no metal in the OM in the case of 5i3e), but extra metals not in the OM were identified (32 or 46%); and class III, where the OM metals and PIXE data agreed (3 or 4%). Class III included both direct agreement and cases where the modeled metal was below the detection limit of the PIXE experimental conditions, and for which no contradictory metal was indicated. For these data, there was no class IV, for which the protein sample was too dilute for successful PIXE measurements. For the NESG samples, 25 out of 28 (89%) had PIXE results that were inconsistent with the OM. Of these, 11 could be explained by a metal present in the crystallization conditions. Two of the samples, proteins 3 and 16, had Mg in the model, not detectable in the experiment, but present in the crystallization conditions, and were classified as class III. For the SSGCID samples, all had XRFS and PIXE results that were inconsistent with the OM, 20 (48%) of these being explainable by a metal incorporated from the crystallization conditions, but not present in the precrystallization sample. The criteria for determining inconsistency were stringent; for example, 4pcl had Mn present in the OM, but Mn was only seen at <2 × LOD in the PIXE data, similarly 3mx6 had Mn present in the OM, but only at <3 × LOD in the PIXE and XRFS data. These two cases were categorized as the experimental data being inconsistent with the model.

### 3.1 Analysis of the Experimental X-ray Data Associated with Metalloprotein Targets

The majority of the NESG proteins incorporated selenomethionine (Se-MET) for phasing purposes with the exception of PDB IDs 1ydn, 2nvv, 4fj4, and 3sm3. Copper (Cu) was also detected in all the NESG samples except 1ydn and is believed to be from material in the beam path during experimental PIXE run 2. Cu was therefore treated differently in the tabulation of metals present, with samples 2rjb and 3guw having 3× the average Cu signal across all samples in this run, and 2gwg, 2× the average signal. The selenium presence is noted in Table S1, but the low-level Cu contaminant is not. There were intense signals for sulfur due to its presence in both the protein and buffer, and also for the chlorine in the buffer. The results for the NESG proteins are given in Table 1. The elemental content was calculated based on the known Se-Met content, as in our previous study.^17^

The results for the SSGCID proteins are given in Table 2: these samples did not have incorporated Se-Met. The Cu beam path contaminant was not present in the SSGCID sample data (runs 3 and 4) so the relative amount of Cu present in the proteins could be assessed. There is agreement between the PIXE and XRFS data for the most significant signal present for 26 samples (including signals that were weaker than 3 × LOD), with another 13 concurring by having the highest signals within the top three metals, yielding over 93% agreement overall. Of the three remaining samples, 3r4t had Ca in the PIXE data but no metal in the XRFS data, 3u0f had Ca and K present in the PIXE data, with Ni present in both the PIXE and XRFS data at <2 × LOD, but only Na present in the OM (presumably from the crystallization conditions), and 3u0a had Na in the model. This latter sample, 3u0a, was used as a control with no metal seen in either the PIXE or XRFS data. The 93% agreement for the 42 samples studied by both PIXE and XRFS is not unexpected, given the similar physical processes involved. For this comparison, the PIXE data represents the signal above background corrected for target thickness, and not the stoichiometric amount calculated based on the ratio of the S or Se concentrations to the number of S or Se atoms in the primary sequence, and the XRFS ratio is directly calculated from the fitted peaks. In the latter case, the integrated area under the peak is converted to a molar ratio using the measurements from the standard foils, which is used for the SSGCID sample results when comparing them to the OM.

### 3.2 Detailed Exemplars of the Experimental Outcomes

In order to illustrate the impact of correctly identifying the metal in metalloprotein structures, we selected two cases for in depth study, and these are described below.

The first example is from the NESG samples: PDB ID 3mel is a structural model of a thiamin pyrophosphokinase family protein from *Enterococcus faecalis*. This protein is essential for central metabolic processes, including the formation of acetyl CoA from glucose and as part of the Krebs cycle. The OM has four chains, with four Mg ions in the first, three in the second, and two in the third and fourth. One of the Mg positions is conserved in all the chains, another two Mg ions are conserved in three of the chains (A, B, and C), while the other Mg ion is conserved in only two chains (A and D). The Mg ion conserved across all of the chains showed positive difference electron density, and the two ions conserved in three of the chains showed significant positive difference density. The PIXE data are insensitive to Mg under the experimental setup used, but indicated the presence of Fe which was not present in the crystallization conditions, Figure 2. Replacing those Mg ions that showed excess positive density with Fe, and then rerefining revealed that the common ion in all four chains fit the density well, but there was still some excess positive difference density for the other two ions present in three of the chains. Replacing these with SO_4_, present in the crystallization conditions, and shifting Arg 55 into a position now seen in the positive difference electron density, further reduced the *R* and *R*_free_ and revealed that the other two Mg ions were likely water. Replacing these with water resulted in *R* and *R*_free_ of 0.168 and 0.249 and an *RSZD* of 2.1 compared to 0.199 and 0.238, and an *RSZD* of 10.8 for the OM. The large *RSZD* for the OM came from one of the Mg ions that was now removed and then replaced by an SO_4_ ion. The equivalent Mg ion for the new *RSZD* of 2.1 with Fe had an *RSZD* of 6.2 in the OM. The CMMS flagged the OM as a problematic case based on metal–ligand distances and geometry. Figure 3 shows the OM with the *2Fo-Fc* and *Fo-Fc* electron densities and the rerefined model using the experimentally determined metal. In addition to replacing the Mg ions with Fe, the correction of the OM with a SO_4_ ion and a remodeled Arg residue also contributed to the decrease in *R* and *R*_free_. The CMMS indicated a far more acceptable fit with Fe. Fe is not known to be involved with thiamin pyrophosphokinases, as a divalent cation, usually Mg, is thought to be needed. Given that the PIXE data indicated the presence of Fe, the positive electron density for Mg in the OM, the CMMS analysis of both the Mg and Fe ions, and the residues coordinating the metal, we conclude that the correct metal in the sample is likely Fe^3+^. Fe^3+^ was modeled, and the PDB deposition updated.

**Figure 2:**
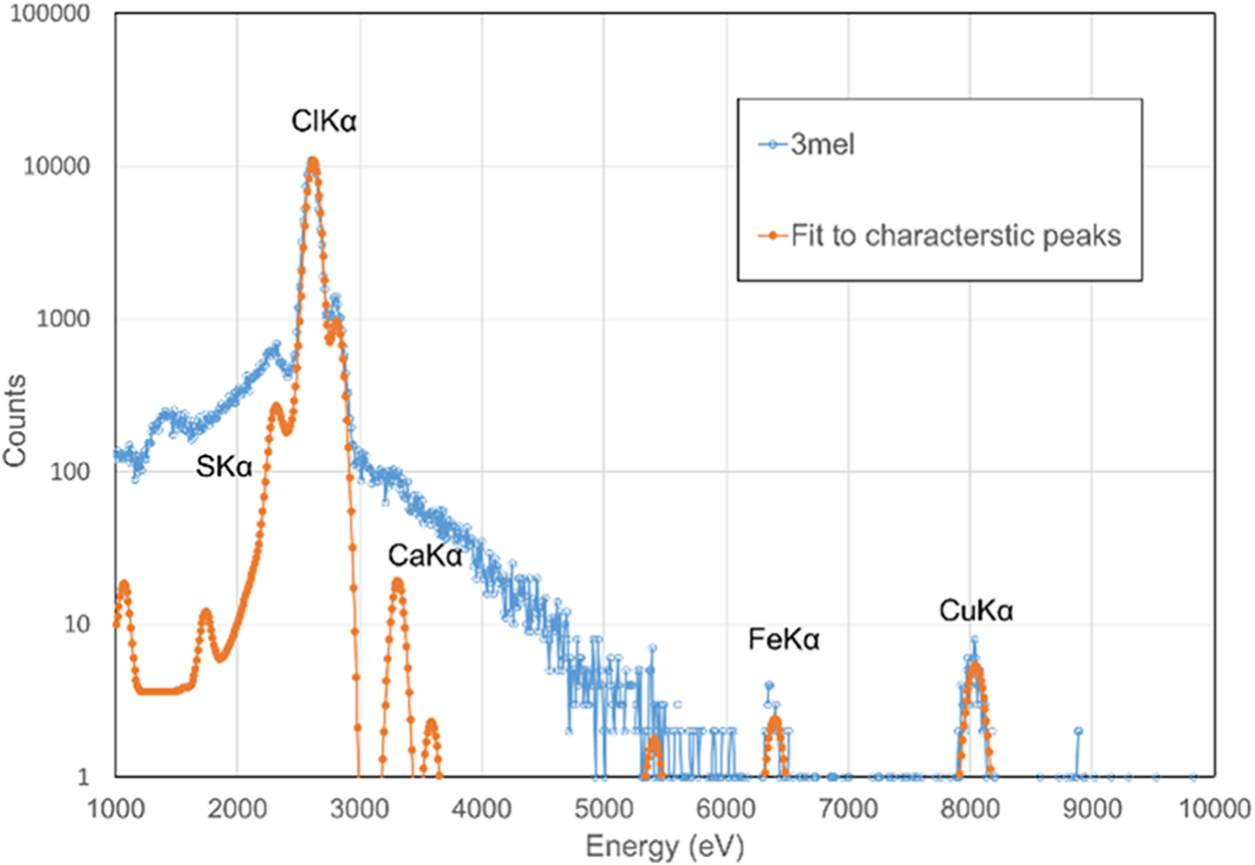
Example of the PIXE spectrum from PDB ID 3mel. The raw spectrum (in blue) is shown together with the background-subtracted fit (in orange) to the spectrum. The Cu signal is from contamination in the beam path.

**Figure 3:**
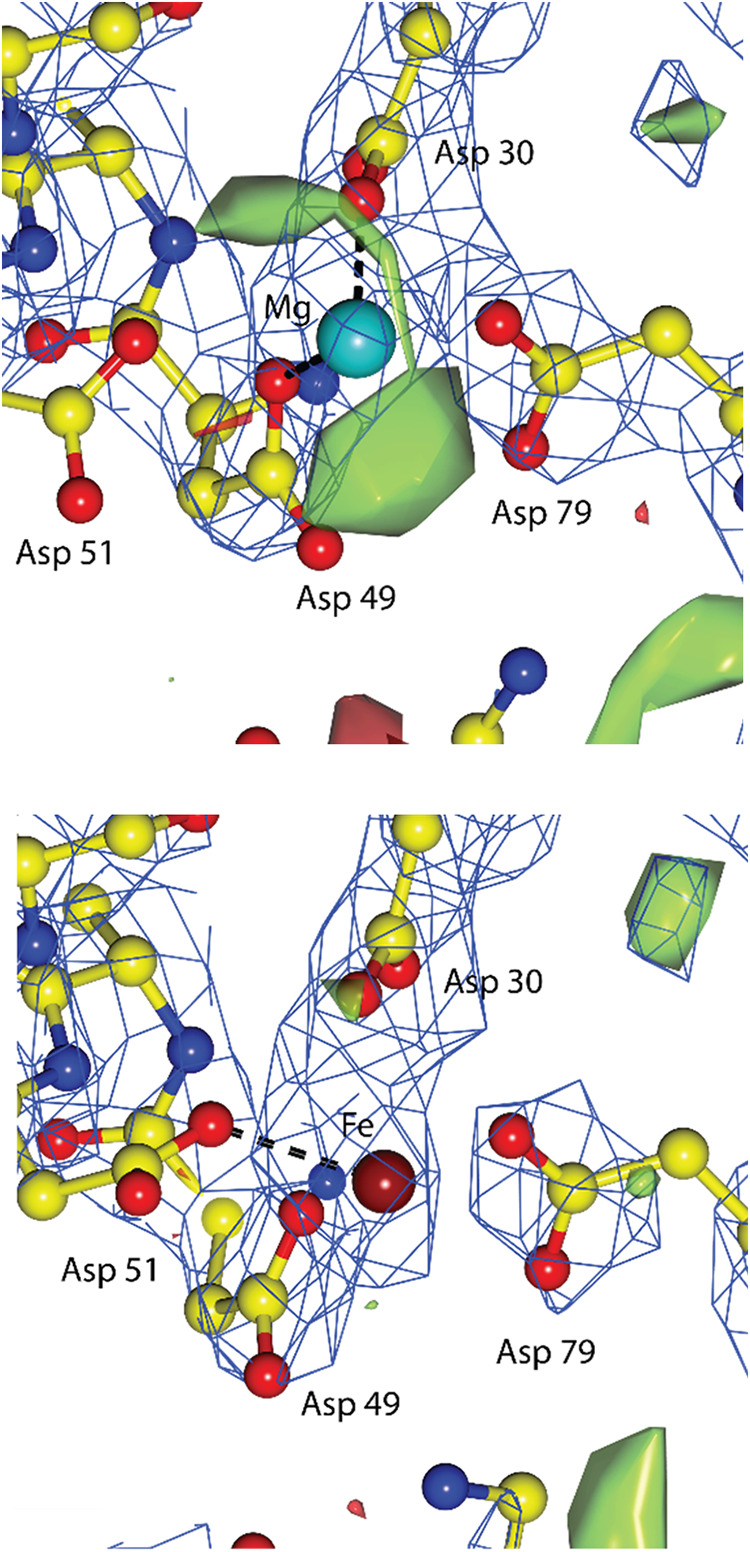
PDB ID 3mel showing the original *2Fo-Fc* and *Fo-Fc* electron density (top) with Mg, and the rerefined model using the experimentally determined metal, Fe (bottom) at 2.79 Å resolution. The blue 2Fo–Fc map is contoured at 2σ, and the Fo–Fc maps are shown as a semitransparent surface contoured at 3σ and – 3σ in green and red, respectively.

The second example, analyzed by both PIXE and XRFS, is one from the SSGCID samples: PDB ID 3mx6, which is a methionine aminopeptidase from *Rickettsia prowazekii* bound to methionine, and is a promising target for the detection of antimicrobial agents.^40^ While there was no divalent ion in the crystallization conditions, in the OM two Mn ions surrounded by waters were modeled in each of the two OM chains, based on analogous models in the literature, *B*-factor similarity, the geometric parameters, and agreement from the CMMS. The difference density was negative, indicating either a reduced occupancy, the absence of the waters, a different metal, or a combination of these possibilities. The XRFS data for this protein are shown in the top half of Figure 4. The images shown are ‘bandwidth’ selected from the full spectrum over an energy range 200 eV above and below the respective fluorescence peak of the element, with the intensity colored from blue as low, to red as high. A strong peak from the concentrated presence of one element can lead to an errant signal for the next higher atomic element due to the K_β_ emission from that peak extending into the bandwidth of the other. The full spectrum for each individual pixel is summed with the spectra of other pixels in each drop profile and averaged to produce the single spectrum shown in Figure 5, corrected for the fluorescence efficiency as a function of the incident X-ray excitation energy. The visible variation in the shapes of the dried drops seen in Figure 4 is due to manual volume differences introduced by deliberately dispensing less than 0.2 μL in some cases, where it was thought necessary to provide an additional visual control to differentiate each protein array. The bottom of the figure shows the data from the protein used to generate PDB ID 4j5i, described later. The metals present in each sample are clearly observed, but the image is colored to maximize contrast and scaled automatically for each fluorescent X-ray energy range. The XRFS spectrum for PDB ID 3mx6 indicated Fe, Ni, and Mn, at normalized signal ratios of 0.79, 0.12, and 0.09 respectively, and Zn at >2 × LOD. PIXE data indicated Fe, with Mn also detected at >2 × LOD and the spectrum clearly shows the various metals present. For comparison with the XRFS results, the PIXE spectrum and fit for 3mx6 are shown in Figure 6.

**Figure 4:**
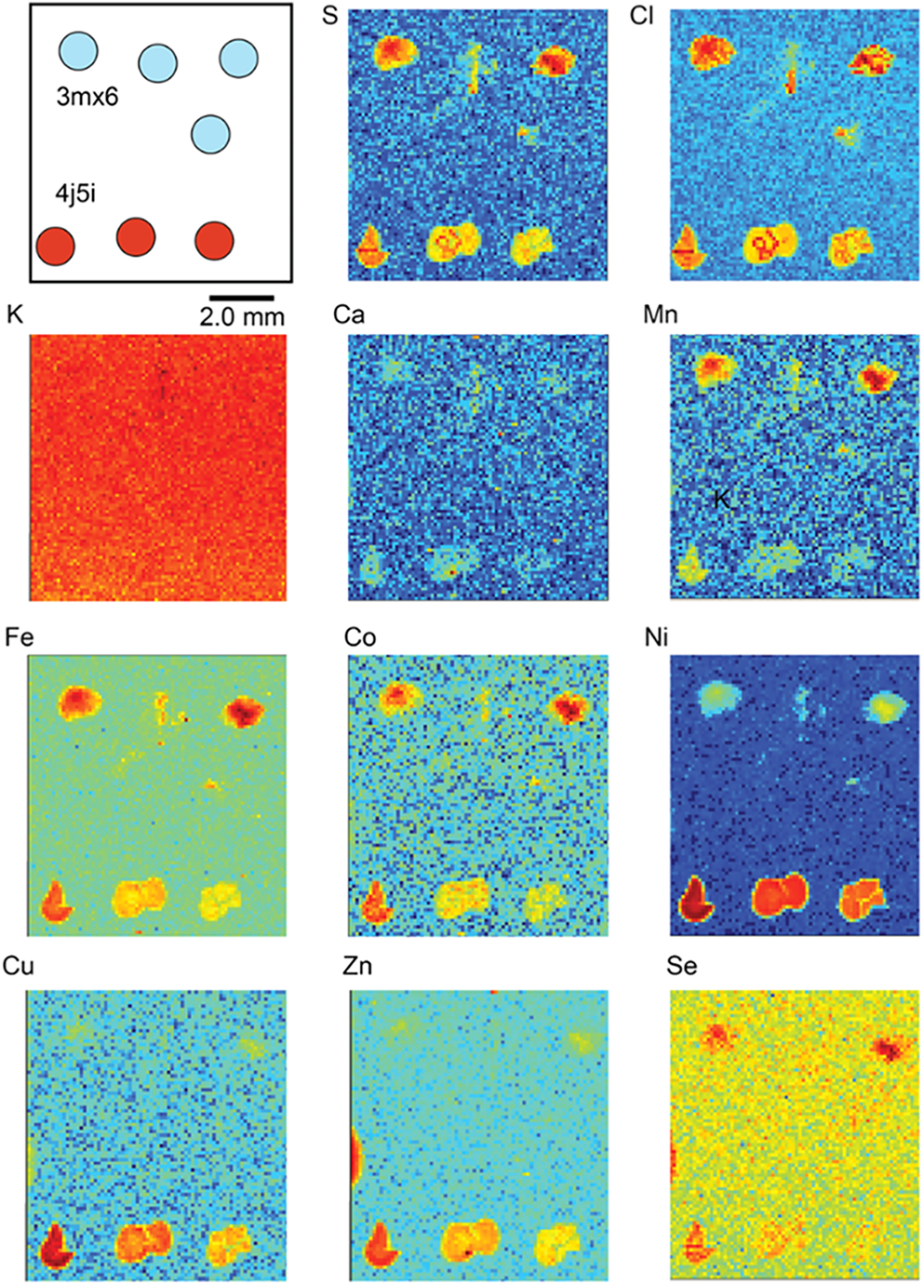
XRFS images for the cassette containing 3mx6 (4 droplets at top of each elemental box image) and 4j5i (3 droplets on bottom row of each image). Shown are a schematic of the drops deposited, and the software defined X-ray windows for S, Cl, K, Ca, Mn, Fe, Co, Ni, Cu, Zn and Se. The colors are presented to illustrate the maximum dynamic range in the data presented here on a log scale, with the raw signal displayed in Figure 5. The presence of Ni, Mn, Fe, and Zn in the raw signal can clearly be seen for 3xm6, and in addition Zn and Cu for 4j5i. For these window ranges, the K_β_ emission of one atomic number element can extend into the window for the next. The fitted spectrum, Figure 5, is used for quantitative analysis. No K is detected. The scale bar is 2.0 mm in length and the overall window size is 8 mm × 8 mm.

**Figure 5:**
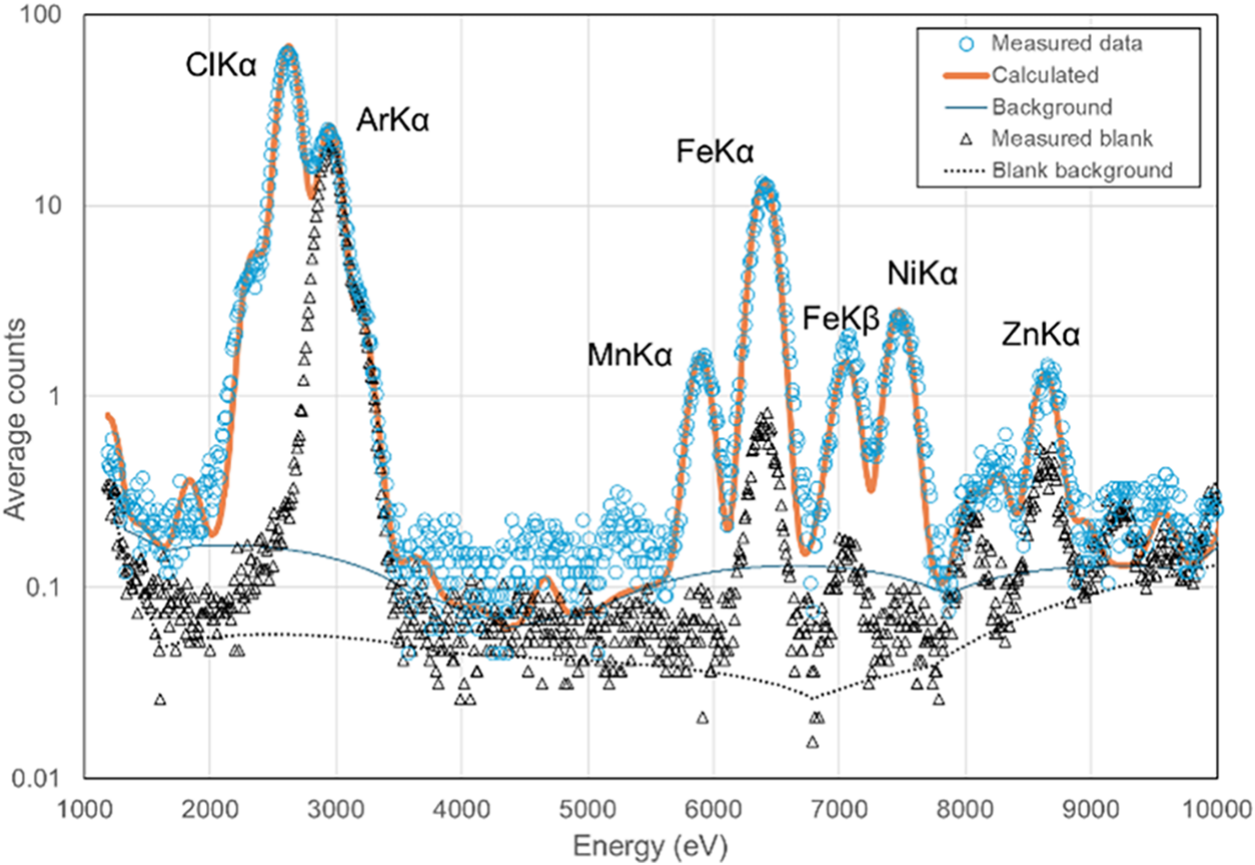
XRFS spectrum for one drop of sample 3mx6. The measured data (light blue circles) along with a calculated fit (orange line), the calculated background (light blue line), and the spectrum of a blank part of the cassette window (black triangles) are shown. Other contributions include argon (ArKα) from the air, pileup (where two photons arriving simultaneously can be recorded as one at the combined energy) and escape events where the detector itself emits characteristic X-rays at an energy of the incident signal less the characteristic detector material emission (SiKα). These are modeled in the calculated fit from PyMCA.^39^

**Figure 6:**
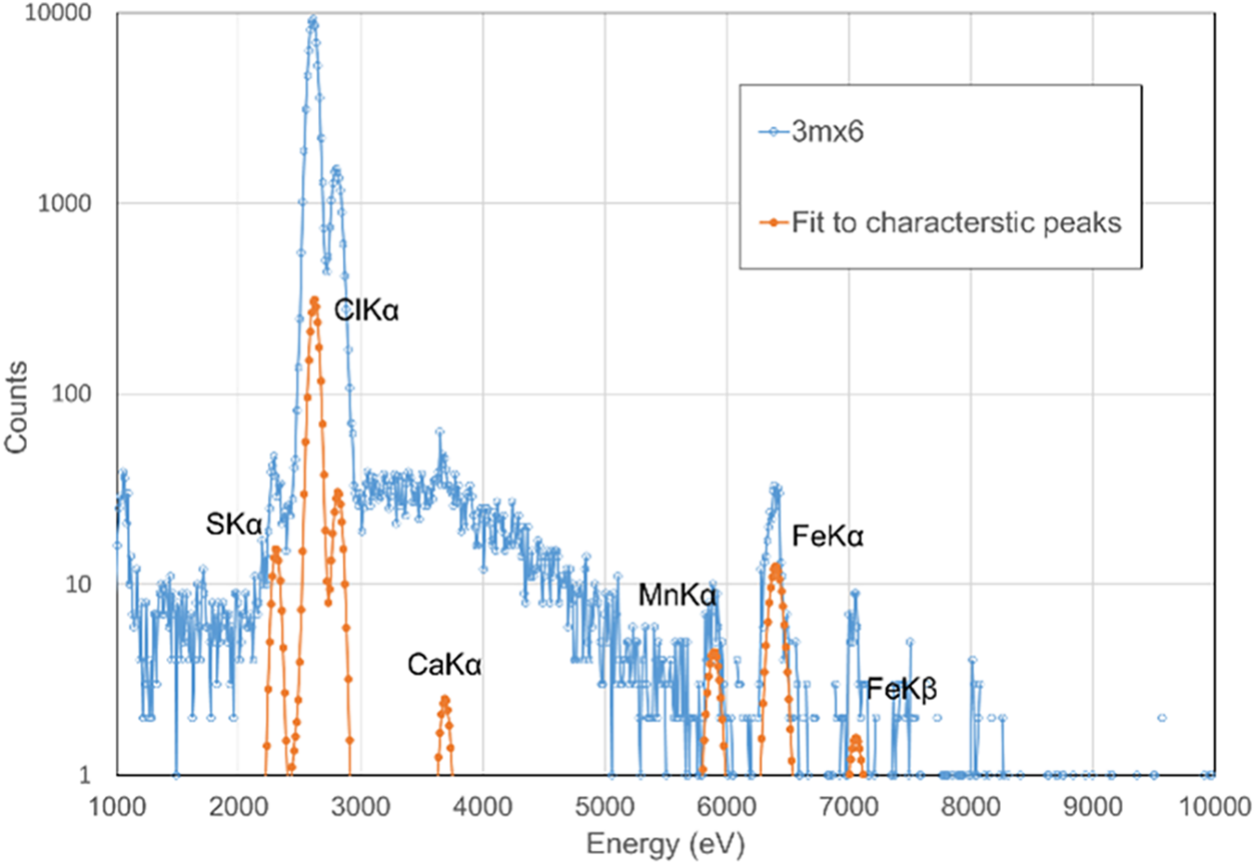
Example of the PIXE spectrum from PDB ID 3mx6. The raw spectrum (blue) is shown together with the background subtracted fit (orange) to the spectrum.

The identity of the physiological metal ions for the dimetal center of methionine aminopeptidase are controversial^41^ but Fe has been suggested as the relevant metal for the *Escherichia coli*. protein.^42^ The Mn in the OM was remodeled with Fe and Ni with no coordinated waters, Na (from the crystallization conditions), and Mg (the negative difference density indicating a lower atomic number metal although our experimental setup was insensitive to Mg). The rerefined models gave an *R* and *R*_free_ of 0.160 and 0.199 for Fe, 0.161 and 0.120 for Ni, 0.167 and 0.205 for Mg, and 0.164 and 0.206 for Na with the OM having 0.170 and 0.203 respectively. The Fe^3+^ ions modeled show no difference electron density, Figure 7, and are clearly supported by both the PIXE and XRFS data. Thus, for methionine aminopeptidase from *R. prowazekii*, Fe appears to be the physiological metal and the new *RSZD* was 0.0 compared to the OM value of −5.7.

**Figure 7:**
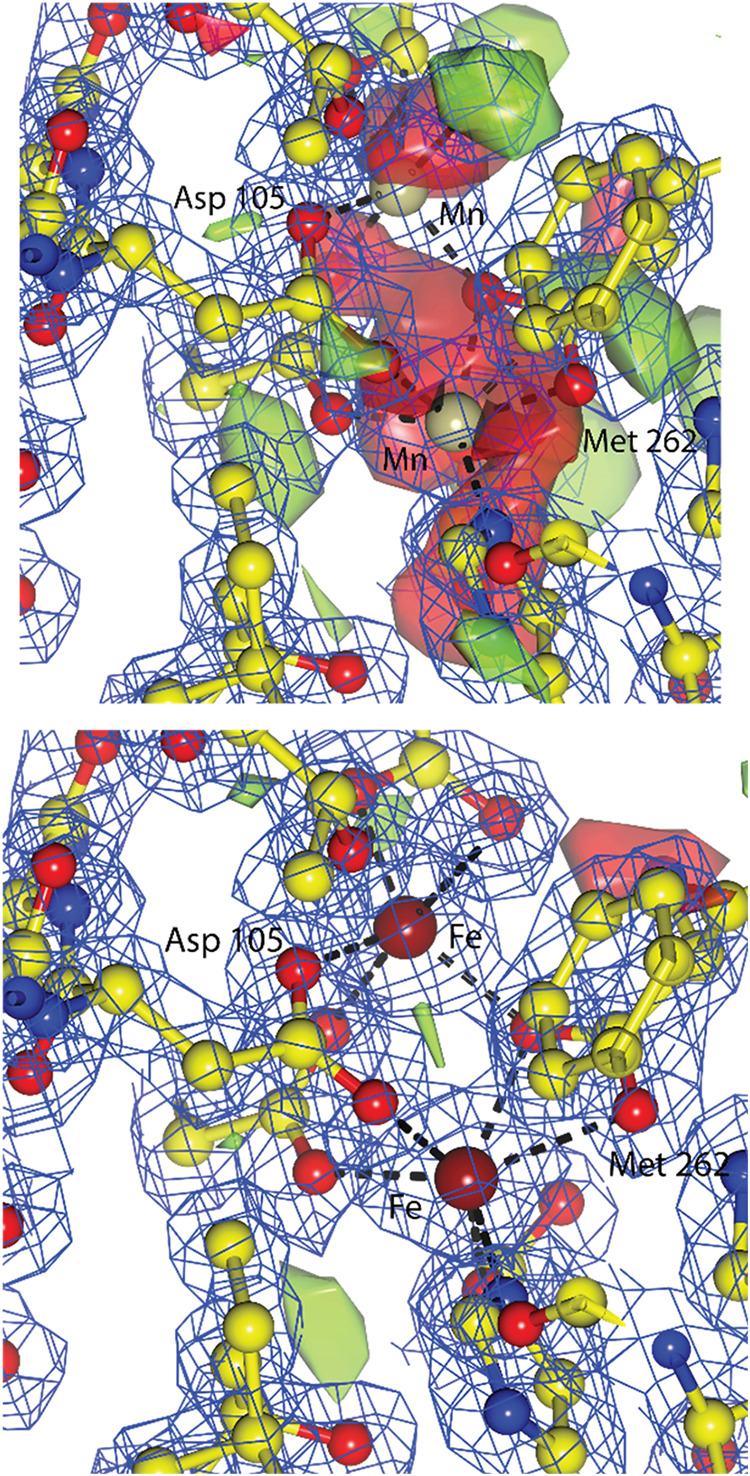
PDB ID 3mx6 showing the *2Fo-Fc* and *Fo-Fc* electron density at 1.70 Å resolution, and the original (Mn) and rerefined (Fe) model using the experimentally determined metal. Similar to Figure 3, the blue *2Fo–Fc* map is contoured at 2σ, and the *Fo–Fc* maps are shown as a semitransparent surface contoured at 3σ and −3σ in light green and red, respectively. The front Fe has a coordination number of 6 and the rear Fe a coordination number of 5.

The XRFS and PIXE data clearly indicate that the metal is Fe, and the CMMS found this was borderline to acceptable. The PDB was redeposited, updating the OM.

### 3.3 Summary of Results for the Other NESG Proteins

The structural analysis results for the 28 NESG proteins are described in the Supporting Information and summarized in Table S3. For these NESG samples, the experimentally identified metals have been placed in the OM and then rerefinement carried out. Any further refinement beyond this after the new electron density was calculated is noted. In some cases, while a different metal from the OM was identified, these metals were not at 100% occupancy, or a mixture was present. REFMAC5 ^43^ was used to refine the occupancies, and then the PDB-REDO process was used with those occupancies. PDB-REDO does not refine occupancy as part of its usual pipeline. Table S3 provides the original structural metrics, the results of the rerefinement, and an analysis of the difference. In this case, the change in *RSZD* is noted as the magnitude of the difference in the magnitude of the original and new experimentally deduced *RSZD*, to allow the shift in the difference electron density toward zero to be seen. In most cases the improvements seen in *R* and *R*_free_ are minor when the experimentally determined metal is modeled, and discrimination of potential metals is poor (described in more detail in the Supporting Information). There was one case where *R* increased slightly (2bdr) and several more for which there were small increases in *R*_free_ compared with the OM (3mel, 2h28, 2bdr, 3c5m and 3sm3) but the differences were minimal and could be explained by an improvement in some general unmodeled detail in the electron density maps triggered by better overall models. High *RSZD* values are the strongest indicator of incorrectly modeled metals and the reduction in these is significant and improved in every case. The goal for a well-modeled metal is a zero *RSZD*, i.e. no positive or negative difference electron density in the *Fo-Fc* map.

There are a few cases that are useful to look at in detail, in particular two samples with copper signals higher than that caused by the background contamination in the NESG run 2 PIXE experiments. The first of these is PDB ID 3guw which is a structural model of the TatD-like Protein (AF1765) from *Archaeoglobus fulgidus*. There are four chains in the OM, with two Zn ions in each chain. In each of the chains there is positive difference density surrounding the same one of the two Zn ions present. The positive difference density would indicate more electrons than are present in Zn. The PIXE data do not indicate the presence of any metal at >2 × LOD but the Cu signal was significantly higher than the average Cu signal seen for all the run 2 samples. The R and Rfree for the OM were 0.243 and 0.321 and replacing the Zn ions with Cu still showed strong excess positive electron density. This was modeled as a third Cu for a trinuclear Cu site yielding an R and Rfree of 0.242 and 0.315 and good agreement with the electron density, and giving an RSZD of 2.0 compared to 6.7 for the OM.

Despite the improved crystallographic agreement, Tat-D type proteins are not known to utilize Cu and are typically associated with divalent metal ions such as Mg^2+^, Mn^2+^, or Zn^2+^. A study on a TatD like DNase from *Staphylococcus aureus* showed that Mg was required for activity but displayed a dinuclear site with a phosphate ion occupying the third position.^44^ No phosphate was present in the crystallization conditions used for the 3guw structure and no P was detected by PIXE. Trinuclear Cu proteins typically play roles in electron transfer, substrate oxidation, and dioxygen activation, which are not the typical functions of TatD unless it has a role in cross-linking pili subunits via oxidative modification or to facilitate energy-dependent processes. The CMMS diagnosed outliers in metal–ligand distances and geometry for the Zn case, and Cu was one of the proposed metals. However, the resolution of the OM is relatively low, 3.2 Å, and despite the lack of a Zn signal, and the excess Cu signal, the proposed trinuclear Cu arrangement remains tentative and will require confirmation through higher-resolution crystallography, anomalous diffraction, or complementary spectroscopic methods before a definitive assignment or biological interpretation should be made.

A second sample with a higher-than-average Cu signal was PDB ID 2gwg, which is a structural model of 4-oxalomesaconate hydratase, LigJ, from *Rhodopseudomonas palustris*. It is involved in the degradation of aromatic compounds, particularly in the protocatechuate 4,5-cleavage pathway. The OM has two chains with a Zn ion modeled in both. In each case there is negative difference electron density in the vicinity of the metal ions, and positive density also present for one. LigI from the soil bacterium *Sphingobium sp.* is known to have Zn in the active site.^45^ However, the PIXE data do not show the presence of Zn, but the crystallization conditions contain Na and Li. The *R* and *R*_free_ of the OM were 0.160 and 0.183; with Na and Li modeled instead of Zn, they were 0.159 and 0.182, and 0.168 and 0.191, respectively, but the electron density did not support either Na or Li. The CMMS flagged the ligand–metal distances as outliers and Cu was proposed as one of the top alternative metals. Cu was then modeled into the structure and refined, giving *R* and *R*_free_ of 0.159 and 0.182 with an *RSZD* of −4.2 compared to the OM at −5.1. With Cu in place, an acetate ion was seen close to each Cu ion. Modeling these and rerefining gave an *R* and *R*_free_ of 0.152 and 0.187 with an *RSZD* of −3.6. We conclude that the metal is Cu and the model was not refined further.

Finally, PDB ID 4jc0 was a unique NESG case. This is a structural model of *T. maritima* holo RimO in complex with pentasulfide.^46^ The reported *RSZD* was low, with the highest value being from an Na ion in the protein, but an Fe cluster in each of the two chains was not initially recognized by Alchemy due to a nonstandard residue name. These Fe ions both exhibit significant positive electron difference density. PIXE data indicate K and Ca ions but show no evidence for Fe, although it is present in both the fermentation and crystallization conditions, and is confirmed in the sample by spectroscopy. It is not clear why there is significant positive difference density, with an *RSZD* of 2.6, but it is noticeable that the two Fe–S clusters have *B*-factors that are approximately eight times that of the ligands to which the cluster is connected. Fe–S clusters can be sensitive to oxygen degradation, and the crystallization did not take place under anaerobic conditions, but this would result in negative electron density rather than the positive observed. The Fe–S clusters are highly electrophilic, so photoreduction may be occurring and this could explain the positive electron difference density.^47^ There was no experimental evidence for any heavier metal, there is an absolute requirement for the two Fe–S clusters in the C–H and C–S bond conversion reactions,^48^ and the model was not refined further.

The structural analyses for the other 24 NESG proteins are detailed in the Supporting Information.

### 3.4 Summary of Results for the Other SSGCID Proteins

The structural analysis results for the 42 SSGCID proteins are also provided in the Supporting Information, summarized in Table S4.

As was the scenario for the NESG samples, in most cases the *R* and *R*_free_ for the SSGCID proteins only show minor changes (*R* increases for 5uf2, and 4hdt, and *R*_free_ is higher for 5uf2, 4y0e, 6ao1, 4wok, and 3s6l). The *RSZD* again proved to be the most sensitive parameter and the |*RSZD*| improved in each case. It should be noted that in a number of cases, occupancy refinement was used. This was guided by the experimental XRFS data but is also an efficient way of reducing the positive difference density that can be explained by a lighter atom or reduced occupancy.

Some specific examples of interesting cases include PDB ID 4pcl, the structure of an O-methyltransferase required for infection of tick cells by the bacterium *Anaplasma phagocytophilum*.^49^ The protein is modeled with Mn but the experimental XRFS and PIXE data show no Mn above the LOD. The XRFS data show, Zn, Cu, and Ni present at >1.5 × LOD and PIXE indicates Ni and, Fe at >2 × LOD but no Zn or Cu. Agreement between PIXE and XRFS is strongest for dominant elemental signals and at lower LOD levels, discrepancies can occur. There are two chains in the OM, both of which contain a single Mn atom with significant negative electron density. Although Mn is not listed in the crystallization conditions deposited in the PDB, the enzyme was incubated with MnCl_2_ before setting up the crystallization.^49^ Zn, Ni, and Cu were modeled based on the XRFS data even though the atomic numbers are higher than Mn. This resulted in *R* and *R*_free_ of 0.166 and 0.199 for Zn, 0.142 and 0.192 for Ni, and 0.160 and 0.200 for Cu, compared with 0.171 and 0.197 for the OM. The respective occupancies of the metal sites in each chain were 84% for both Zn ions, 100% for Ni, and 90% for Cu. The resulting *RSZD* values were −5.2 for Zn, −3.5 for Ni, and 4.2 for Cu. The remodeling revealed a partial water coordination sphere around the ions and a movement of a residue in each site away from direct contact with the metal and forming a water mediated contact. Despite the MnCl_2_ incubation, the metal appears to be Ni. The original *RSZD* for Mn was −12.5. No further refinement took place.

Another challenging case is PDB ID 4y0e, a putative dioxygenase from *Mycobacterium abscessus*, a multidrug resistant bacteria that can cause diseases in almost all human organs.^50^ It was modeled with Fe present, but this was not seen in the experimental data and it was not present in the crystallization conditions. There are eight chains in the OM with one Fe ion in each chain, all with negative electron density indicating too high an atomic number element modeled, and/or a lower occupancy than modeled. The XRFS data indicate Ni and Zn, in the ratio of 0.53 and 0.47, and Ca present at >2 × LOD. The PIXE data also show Ni, with Zn seen at >2 × LOD. Mg was present in the crystallization conditions together with Ca and Na. Ni has been observed in dioxygenases^51^ and it is likely that Ni was sequestered during the expression and purification process but is present at low occupancy. The Fe ions were replaced with Mg and Ca, and each model rerefined. The Mg ions resulted in an *R* and *R*_free_ of 0.175 and 0.201, and the Ca, 0.178 and 0.202, compared to the OM at 0.177 and 0.202. Ni and Zn were modeled at a reduced occupancy of 50%. The *R* and *R*_free_ for Ni ions at 50% occupancy was 0.165 and 0.204 and there was little to no difference density associated with the ion positions with an *RSZD* of −3.4 compared to the OM at −11.3. REFMAC5 was used to refine the Ni ion occupancies and the model rerefined. The *R* and *R*_free_ were 0.156 and 0.207 with an *RSZD* of 2.3, and thus we conclude metal in the model should be Ni.

Finally, PDB ID 4yet is a structural model of superoxide dismutase from *Babesia bovis*. It is modeled as two chains with an Fe ion in each chain.^52^ 4yet was a “potential drug target” nominated by the Tropical Disease Research (TDR) targets consortium.^53^ Fe ions were modeled on the basis of interatomic distance values, with the CMMS indicating acceptable occupancy, *B*-factors, elemental composition of the coordination sphere, bond valence values, and deviation of the observed geometry compared to the ideal geometry. The summation of ligand vectors and arrangement of the ligands around the ion are regarded as borderline. The Fe model has an *RSZD* of 3.3. The XRFS data show Fe, Mn, Ni, Se, and Zn at >3 × LOD with normalized ratios of 0.68, 0.17, 0.10, 0.02, and 0.02, and Co present at >2 × LOD, a similar result to the PIXE data which indicates Mn and then Fe. Fe is present in the crystallization conditions. Rerefining with Mn replacing the Fe ions yields *R* and *R*_free_ of 0.143 and 0.171 with no difference density seen around the Mn positions. The OM had *R* and *R*_free_ of 0.151 and 0.173. The *RSZD* is reduced to 1.5 from 3.3 for the OM. Refining with a mixture of Fe and Mn in each site resulted in occupancies of 0.75 and 0.25, and an *R* and *R*_free_ of 0.125 and 0.168, with an *RSZD* of 0.5. These new data demonstrated that the superoxide dismutase from *B. bovis* is better modeled as a mixed MnSOD and FeSOD. Interestingly, an analysis of several superoxide dismutase structures including PDB ID 4yet assuming Fe ions, notes similarity to human Mn superoxide dismutase.^52^ The Fe structural discussion is further detailed in the crucial role of iron acquisition in *Leishmania*.^54^ While the model is improved with Mn, it is much improved by a mixture of Fe and Mn in agreement with the XRFS and PIXE data.

These examples demonstrate the incorporation of a promiscuous metal from the crystallization conditions, the Ni presumably being aquired during the purification process, and the misidentification of Fe for a mixture of Fe and Mn, causing the propagation of mis-identified Fe in the literature. If we exclude cases for which Ni was the only experimentally detected contradictory metal, only PDB entry 4o5o would be reclassified, so the overall results are only minimally impacted by Ni incorporation during purification. Further details of the structure remodeling for the other 38 SSGCID proteins are provided in the Supporting Information.

## 4 Discussion

While errors in ligand modeling or side chain placement are well recognized, metalloproteins appear to represent a particularly vulnerable class of structures because metal substitution can occur adventitiously during expression, purification, or crystallization. Once incorporated, metals with similar scattering properties may not be readily distinguished by crystallographic analysis alone. The analyzed protein models were produced by large-scale structural initiatives whose work to produce the best quality structural information enabled our experiments to be carried out. The quality of structural models produced by such efforts has been analyzed and shown to be equal if not better than those produced by individual laboratories in many contexts.^55,56^ Our investigation into the identity of metals in 70 different metalloproteins studied confirmed the conclusions of our previous PIXE study^17^ for which 17/30 metalloproteins had the incorrect metal built into their PDB model. The sets of proteins described in the current study include suspect and also good RSZD scores for the metals in the OMs. Inspecting the RSZD cutoffs, Table S1 (NESG) and Table S2 (SSGCD) show 11 NESG samples with an RSZD > 6, 14 at <6, and 3 at zero with no metal present. For the SSGCID set, 20 proteins had an RSZD > 6, another 20 had values <6, and 2 were at zero, with no metal present.

To assess the relationship between RSZD and experimentally validated metal assignments, we evaluated its diagnostic performance using a receiver operating characteristic (ROC) analysis^57^ at thresholds of 3 and 6 with our classifications. Proteins classified as Class I (PIXE/XRFS inconsistent with the original model) were treated as positives, whereas Class III proteins (agreement between PIXE/XRFS and the original model) were treated as negatives. For proteins containing multiple metal sites, the highest absolute RSZD value associated with that entry was used, consistent with the classification scheme used throughout the study. Class II proteins where the experimentally detected metals included those that were modeled in the original structure but also indicated additional metals, were excluded because they represent partially consistent rather than clearly correct or incorrect assignments.

Using this definition, the analysis was performed on 37 proteins consisting of 34 positive cases (18 NESG Class I and 16 SSGCID Class I entries (5i3e with no metal present)) and 3 negative cases (Class III entries). At a threshold of RSZD > 3, the sensitivity was 0.71 (24/34 incorrect cases identified), indicating that the majority of experimentally inconsistent metal assignments were detected. However, all three Class III proteins also exceeded this threshold. Increasing the threshold to RSZD > 6 reduced the sensitivity to 0.50 (17/34 incorrect cases identified) but improved the specificity to 0.67 (2/3 correctly assigned cases identified). Because only three Class III proteins were available, the specificity estimates should be interpreted cautiously.

The limited discrimination observed in the ROC analysis is consistent with the broader experimental findings. Elevated RSZD values occur not only in proteins whose modeled metals are inconsistent with PIXE/XRFS measurements (Class I), but also in proteins where additional experimentally detected metals are present (Class II). Thus, RSZD appears sensitive to crystallographically anomalous metal sites in general rather than being uniquely attuned to incorrect elemental assignments. Consequently, RSZD is best interpreted as a screening metric that identifies sites warranting further investigation using complementary approaches such as PIXE, XRFS, anomalous diffraction, or detailed crystallographic validation. Nevertheless, the observed association between elevated RSZD values and experimentally identified metal-site anomalies provides experimental support for the use of crystallographic validation metrics in prioritizing suspect metal assignments for further analysis and large-scale database curation.

Extrapolating our findings to the >40,000 metalloproteins currently in the PDB presents a significant challenge. To provide context we analyzed ∼160,000 metal sites in the PDB using the CCP4^29^ program EDSTATS.^30^ Of these, over 20,000 metal sites exhibited an RSZD exceeding 6, and over 56,000 of them had RSZD values exceeding 3. Focusing on the highest 5,000 RSZD values (11.2 and greater), we examined their origins to determine whether they were produced by structural genomics (SG) centers (data provided in the Supporting Information). A total of 373 entries (∼7.5%), originated from SG centers, compared to an expected baseline of 5% of PDB depositions, indicating a modest ∼1.5% enhancement. Thus, while SG-derived structures are somewhat overrepresented among the most extreme RSZD outliers, the vast majority (>90%) of these cases arise from non-SG efforts. This distribution likely reflects differences in experimental strategy: dedicated metalloprotein studies often benefit from system-specific biochemical and spectroscopic validation, whereas high-throughput pipelines necessarily prioritize scale and uniformity. Importantly, the discrepancies we observe in both SG (27/28 NESG and 42/42 SSGCID samples) and non-SG data sets emphasize that challenges in accurate metal assignment are widespread, and that our PIXE and XRFS approaches are broadly applicable across both contexts.

Both the PIXE and XRFS data rely on similar physical processes (electronic level excitation followed by X-ray emission) and would be expected to be in agreement. In the case of the SSGCID samples for which we could use both the XRFS and PIXE techniques, the highest three metals identified are consistent for over 93% of the samples. However, the PIXE analyses could not be quantitative due to the presence of sulfur in the protein buffers. In PIXE measurements on proteins, sulfur (or phosphorus for DNA and RNA) is the element usually used for internal normalization to obtain stoichiometric values for metal atoms per protein molecule to within ±10%.^15^ Here, a different method had to be adopted, and ratios of the metal signals were used to make quantitative estimates from the PIXE data, normalized to the sum of signals >3 × LOD. In the XRFS case, the fluorescence is fitted quantitatively with the measure of the incident flux, knowledge of detector parameters, and known physical properties of the elements^39^ and the ratios were converted to molar ratios. As carried out for the PIXE data, the measurements were normalized to the sum of signals >3 × LOD. The data from both techniques are very consistent. Because both methods only detect the elements present, the state of the sample is of no concern. PIXE run 2 was carried out in 2019 and runs 3 and 4 in 2023. The XRFS data were collected in 2024. The sample cassettes were stored at ambient temperature between studies. The atomic composition of the samples used in both methods were stable over this period. The two techniques, PIXE and XRFS have differences in excitation mechanisms, detection efficiencies, and limits of detection. Despite this, the different approaches yielded largely consistent, robust results.

Our study establishes PIXE and XRFS as reliable and largely congruent methods for elemental identification. Both techniques can be applied retrospectively to archived samples, even years after initial crystallization, extending their utility for community-wide reanalysis of deposited models. The techniques add new information that enabled the accurate identification of the metal present in the original protein sample used to grow the crystals for X-ray crystallographic model determination. We do not probe physiological metalation but rather test whether the metals modeled in deposited structures are consistent with the elemental composition of the protein material used to generate those structures. PIXE and XRFS are not without limitations. Neither method provides information on oxidation state or site occupancy, nor can they resolve mixed populations of metals at a single binding site or distinguish between bound from unbound metal in the solution. Our conclusions are therefore restricted, and establishing the physiological metal and metalation state still requires targeted biochemical investigation.

In some cases, where different or additional metals have been detected by PIXE and XRFS, published literature giving other experimental biochemical evidence of a protein’s metal identity has clearly supported the likelihood of one metal above another. Where such information is unavailable, we cannot postulate whether the measured metal is the native one, or another one acquired through promiscuous incorporation in an experimental step during the protein production or purification. We do have information on the metals present in the crystallization conditions, and when those are seen in the model but not in the precrystallization sample, we can thus be more certain of their origin.

The majority of the NESG samples were produced in *E. coli* systems with SeMet included for phasing purposes. Because we know the protein sequences and characterize the samples with mass spectrometry as part of the production pipeline, we have confidence that for proteins expressed in bacteria all the SeMet sites are incorporated, the concentration of Se is defined. Thus, the accurate concentration of the metals in the sample can be determined from the metal to Se ratios. However, for the majority of the SSGCID samples, Se was not present, and the S signal was swamped by the sulfur in the buffer although through the XRFS measurements and calibration with metal standard foils, we were able to calculate the concentrations of the metals. While this is of some utility, there are issues that complicate absolute analysis. For example, the quantitation is dependent on knowing first the estimated original protein concentration which can have a significant error, and second the volume of the sample deposited on the holder is also required. For these reasons, we have calculated the concentrations in terms of the LODs and used these to determine the ratios of metal concentration in each protein sample. Normally for the PIXE experiments, neither the protein concentration nor the deposited volume is required to obtain the metal stoichiometry to ±10%. For the SSGCID samples, Ni is frequently detected. The protein purification workflow for these samples includes an extra nickel-based metal affinity chromatography step.^35^ Ni is not commonly found in eukaryotes, but is widely used by prokaryotes.^58^ While we can detect a Ni signal, we cannot determine if the Ni is introduced from the purification process, or if it is natively present. However, the systematic presence of Ni in the SSGCID samples having a second exposure to Ni in the purification process, contrasted with its absence in NESG samples, strongly suggests that purification methodology alone can imprint a detectable and potentially misleading metal signature on structural biology samples. The presence of Ni should not necessarily be interpreted solely as an experimental contaminant. In several cases, the crystallographic refinement metrics and electron density are more consistent with Ni than with the originally modeled metal, indicating that Ni was incorporated rather than being solely due to nonspecific contamination. Our primary focus is the reliability of the deposited structural models rather than the biological relevance of the incorporated metal itself. In such cases, the experimentally observed metal occupying the site may differ from the metal reported in the deposited model, even when the substituted metal was introduced during purification and was not present in the crystallization conditions.

PIXE is a well-established technique for measuring metal identity and content in proteins, and we have shown here that the XRFS method has the sensitivity to provide valuable information. For example, if we take a 100 kDa metalloprotein containing a single Zn atom (65.38 Da) then 0.00065 (0.065%) of the total mass is metal. For a typical crystallization experiment the protein concentration is ∼10 mg/mL and in this case the metal concentration would be 6.5 μg/mL, well above the LOD for XRFS at lower than 0.01 to 0.5 μg/mL, depending on the metal. In this study, calibrating the integrated counts to the standard foils, Zn gave a signal at >3 × LOD for ∼0.02 μg of metal in the 50 × 50 μm pixel area, and K for ∼0.29 μg of metal in the same area. XRFS is thus sensitive to metals at the concentration present in typical protein solutions used for structural biology investigations.

Many studies also make use of model bond length and geometry to improve macromolecular structures. Among others, the CMMS^59^ is a primary example of the use of these parameters for efficient metal validation. Metals have known bond lengths and geometry^60^ but as the resolution of the structure decreases, errors in the estimation of bond lengths increase. For example, as a rule of thumb errors in hydrogen bond distances range from 0.1 to 0.3 times the resolution of the data collected.^61^ Validation of metal sites based on the model alone, rather than on using the primary data from which the model was derived, gave examples of both false positives and false negatives. Our analysis is unique in that we have made use of the experimental data deposited with the models to identify suspect metal identities (the Alchemy script) and performed measurements on the original precrystallization protein samples, used for determination of those models.

The question arises as to the relative weighting that should be given to the various available quality metrics in deciding on the most probable metal in the samples. High *RSZD* values seem to be the strongest indicator of incorrectly modeled metals, and the reduction in these values is significant and improved in every case reported here. The goal for a well-modeled metal is a zero *RSZD*, i.e., no difference electron density in the *Fo-Fc* map, and achieving this often requires occupancy refinement for the metal sites. In the summary tables above, we have reported only the highest *RSZD* for any metal in the model, but *RSZD* values are in fact available for every atom. In this work the highest value has been used as a convenient metric, since it is an automated measure of difference density in the electron density maps. In fact, the analysis of 3mel demonstrates that the RSZD is equally useful for flagging other ions or incorrectly positioned residues. The CMMS analysis also assisted our process, but it should be remembered that its results are based on expectations derived from a subset of metalloproteins in the PDB which may well include suspect cases, such as those uncovered in our study. The *R* and *R*_free_ values are also useful indicators of a model that better fits the diffraction data, but as demonstrated in several cases reported here, improvements in the electron density from more accurate phases due to corrected ion assignments often lead to broader enhancements throughout the model. These refinements, in turn, contribute to improved quality metrics during model optimization. *RSZD* values serve as a localized site specific metric and are more sensitive to mismatch at the metal site than *R* and *R*_free_ values.

The metal occupancy was refined in some cases where the XRFS and PIXE data indicate a specific metal is present, but the electron density did not support full occupancy. The occupancy and *B*-factor are correlated, and stronger scatterers can have anisotropic scattering, even at moderate resolution.^62^ Any brute force approach, where multiple metals could be used in parallel processing strategies, is limited by this relationship unless reliable automated rerefinement is also attempted. Of the 70 proteins analyzed here, the structural rerefinement results and some brief biochemical implications for the changed metal identities for eight selected cases have been described above. The available literature on the particular proteins largely supported the experimental observations. Summaries of the results for the remaining 62 can be found in the Supporting Information.

Our focus in this large-scale study has been to validate the potential for high-throughput XRFS as a more accessible alternative to PIXE for unambiguous metal identity in proteins, rather than to carry out an in-depth analysis of the biochemical implications of the corrected metal identities. XRFS is a technique that is available at many synchrotron sources, and such accessibility provides an advantage over the PIXE technique, although both methods have complementary strengths depending on the questions being asked. In each case, the experimental determination of the metal or metals present in the protein will be the start of the investigation, not the end.

In summary of our study, there was over 93% agreement between the PIXE and XRFS techniques. For the NESG samples, 25 out of 28 (89%) had PIXE results that were inconsistent with the OM. Of these, 11 could be explained by a metal present in the crystallization conditions. For the SSCCID samples, all had PIXE results that were inconsistent with the OM, 20 of the 42 being explainable by a metal present in the crystallization conditions. Given the cases where extra metals were observed in PIXE or XRFS, but the rerefinement of the OM with those metals did not impact the model, and those where the metal has been incorporated from the crystallization conditions, the results from all 70 proteins were very similar to our initial work on 30 proteins in which we found that over half of the metals had been mis-identified in the PDB models. It is thus fair to say that if no specific analyses have been performed to identify the metals present, and if relevant biochemical studies are inconclusive or unsupportive, then the metal identity in a deposited PDB model should be treated with suspicion.

Machine learning and artificial intelligence approaches may offer a path forward to accurate metal identification, but they will be impacted by the errors we have identified in the available training sets. The identification of a significant number of incorrect metals is notable in this context; however, these misidentifications reflect the best models available at the time of PDB deposition. Importantly, the PDB provides not only the structural model but also the experimental data used to derive it, allowing our retrospective evaluation and reanalysis by others as new methods emerge. To maximize the utility of large training sets for machine learning applications, it is critical to understand how these models are obtained from the underlying data to ensure that systematic errors do not propagate. The remediation of PDB metal identities would help this but it requires a substantial experimental effort and the development of a high-throughput validation pipeline. This is an ambitious but feasible task given the availability of samples even years after their initial characterization. The need for future large-scale corrections could be significantly reduced by the routine use of excitation scans, even in cases where no metal is expected, given the high prevalence of metalloproteins and the essential roles metal ions play in protein structure and function.

However, we anticipate future advances that will integrate the availability of experimental diffraction data, additional experimental studies, and detailed model analysis to further enhance accuracy, particularly given the community’s forward-thinking requirement that experimental data be made available alongside the corresponding structural models. While both the PIXE and XRFS methods do not provide any positional information on the metals present, neither require that the sample diffracts and they can both provide unambiguous data on the metals present, supplementing the information that X-ray (or neutron) crystallography, Nuclear Magnetic Resonance (NMR), and cryo-electron microscopy provide. In an ideal situation an X-ray excitation scan could be carried out on a crystal sample after diffraction data collection has finished. Even more effective would be anomalous data collection to determine both the identity and location of metals that are present.^17^ Other techniques are available such as EXAFS that can resolve identity and positional ambiguity. There are also many techniques to characterize presence of metals in proteins during their preparation. Incorrect metal assignment is a widespread issue in deposited metalloprotein structures. While the problem is serious, it is also tractable: PIXE and XRFS provide rapid, high-throughput solutions that can be applied both prospectively and retrospectively. The path forward is clear. By integrating these tools into structural biology pipelines, and by elevating expectations for metal validation in publications and databases, the community can substantially reduce the prevalence of misidentified metals. Doing so will improve the reliability of mechanistic interpretations, the reproducibility of biochemical insights, and the fidelity of data-driven methods built upon the Protein Data Bank.

In summary, our study demonstrates that inconsistencies between modeled and experimentally observed metal content in deposited structures are widespread and not readily identifiable using standard structural validation tools alone.^63^ By combining elemental spectroscopy with crystallographic analysis, we show that discrepancies in metal assignment can be systematically detected and, in many cases, resolved through rerefinement. While PIXE and XRFS provide only elemental constraints and do not address oxidation state, coordination geometry, or physiological relevance, they offer an independent and experimentally grounded perspective on the chemical composition of the samples used to generate structural models. The high level of agreement between PIXE and XRFS further supports the robustness and scalability of this approach. Importantly, these findings extend beyond the specific systems studied here: they highlight a broader issue of chemical data integrity in structural databases that are extensively used in computational chemistry, bioinformatics, and machine-learning applications. Ultimately, this work establishes an experimentally validated link between elemental identity and crystallographic validation metrics, demonstrating that chemically inconsistent annotations can be systematically detected at scale, and underscoring that the reliability of computational modeling and machine learning depends critically on the chemical correctness of the underlying structural data.

## Supplementary Material





## Data Availability

The data underlying this study are available in the published article, its Supporting Information, github.com/snelllab/Alchemy, Zenodo (DOI 10.5281/zenodo.20447322), and through the Protein Data Bank.

## Notes

Metal data sorted (XLSX)
Details of the structural rerefinement for the samples in class I and II (PDF)
