# Supplementary material for: Assessing Metal Ion Assignment Accuracy in Protein Data Bank Models via Elemental Spectroscopy
Source: J Chem Inf Model. 2026 Jun 15;66(15):9057–75. doi: 10.1021/acs.jcim.6c00352 (PMC13418866; doi:10.1021/acs.jcim.6c00352)
Supplement: Supplementary file 1 [file ci6c00352_si_003.pdf]

SUPPORTING INFORMATION

Assessing Metal Ion Assignment Accuracy in Protein Data Bank Models via Elemental Spectroscopy

Edward H. Snell[1,2], Geoffrey W. Grime[3], Samuel M. Webb[4], Catia Costa[3], M. Elizabeth Snell[2], John F. Hunt[5], Liang Tong[5], Gaetano T. Montelione[6], Rachel Zigweid[7], Bart L. Staker[7], Peter J. Myler[7,8] and Elspeth F. Garman[9]

[1]The Department of Materials Design and Innovation, SUNY University at Buffalo, 120 Bonner Hall, Buffalo , NY 14260, USA, [2]The University at Buffalo Hauptman-Woodward Institute, 700 Ellicott St. Buffalo NY 14203, USA, [3]University of Surrey Ion Beam Centre, Guildford, Surrey GU2 7XH, U.K., [4]Stanford Synchrotron Radiation Lightsource, 2575 Sand Hill Road, MS69, Menlo Park, CA 94025, USA, [5]Department of Biological Sciences, Columbia University, New York, NY 10027, USA, [6]Deptartment of Chemistry and Chemical Biology, Center for Biotechnology and Interdisciplinary Sciences, Rensselaer Polytechnic Institute, Troy, New York, 12180 USA, [7]Seattle Structural Genomics Center for Infectious Diseases, Seattle, Washington, USA, Center for Global Infectious Disease Research, Seattle Children's Research Institute, Seattle, WA 98101, [8]Department of Global Health, University of Washington, Seattle, WA 98195, USA, [9]Department of Biochemistry, Dorothy Crowfoot Hodgkin Building, University of Oxford, South Parks Road, Oxford, OX1 3QU, UK.

Experimental

Table S1 details the NESG samples studied by PIXE. Each entry provides the PDB model ID, a description of the protein, the Uniprot ID, the number of residues, molecular weight, metal in the OM, the highest Alchemy derived *RSZD* for any metal in the model, and the presence of any metal in the crystallization conditions used to grow the crystal from which the model was obtained. The proteins are ordered according to the magnitude of the largest *RSZD* and then by their molecular weight from high to low if their *RSZD* values are identical. The metals present in the optimized crystallization conditions are reported in Tables 1 and 2. Run number is the experimental run described in the main text and MSE is the number of Seleno-Methionine residues.

Table S1. NESG samples studied by PIXE.

	PDB ID	Run	MSE	Description	Uniprot ID	Residues (Chains)	d_{max} (Å)	R/R_{free}	MW (kDa)	Metal/s in PDB	RSZD	Crystallization metal/s
1	3vb9	2	16	unknown function	Q87I71	1842 (4)	2.10	0.224/0.274	223.91	Mg	16.2	Mg
2	4py9	2	12	exopolyphosphatase-related protein	Q64PD9	340	2.25	0.231/0.264	40.24	Na	12.4	K, Na
3	3goc	2	1	Endonuclease V (SAV1684)	Q82MH6	456 (2)	1.60	0.177/0.194	50.61	Mg	-12.2	Na, Mg
4	3mel	2	3	Thiamin pyrophosphokinase family protein	Q82ZE3	853 (4)	2.79	0.207/0.275	102.82	Mg	10.8	Na, Mg
5	2f6k	2	7	Amidohydrorolase II	F9US96	612 (2)	2.50	0.192/0.225	69.82	Mn	9.4	Mg
6	3sm3[1]	2	2	SAM-dependent methyltransferases Q8PUK2_METMA	Q8PUK2	212	2.20	0.192/0.246	27.16	Ca	8.5	Ca
7	2gzx	2	6	TatD deoxyribonuclease	A0A0H3JV42	506 (2)	2.20	0.258/0.286	61.58	Ni	7.4	Mg
8	2hf1	2	3	putative Tetraacyldisaccharide-1-P 4-kinase	Q7NSS5	120 (2)	1.90	0.233/0.284	16.07	Zn	7.0	Na
9	3bb6	2	4	P64488 protein	P64488	436 (4)	2.30	0.219/0.261	59.81	Zn	6.9	Na
10	3guw	2	7	TatD-like Protein	O28509	932 (4)	3.20	0.241/0.292	120.28	Zn	6.7	Mg
11	2h6l	2	3	unknown function	O30132	420 (3)	2.00	0.187/0.237	50.74	Zn	-6.4	Na
12	2h28	2	4	unknown function	P76364	213 (2)	2.10	0.22/0.253	30.18	Mg	-5.9	Mg

[1] Corresponding author, esnell@buffalo.edu

13	2bdr	2	7	Putative Ureidoglycolate hydrolase	P59285	332	1.60	0.198/0.218	40.45	Na	5.6	Na
14	2gwg	2	10	4-Oxalomesaconate Hydratase	Q6N0R4	655 (2)	1.80	0.181/0.208	80.24	Zn	-5.1	Na, Li
15	4h9d	2	2	Mn-dependent Gme HNH nicking endonuclease	Q39X46	268 (3)	2.60	0.233/0.251	40.37	Zn, Mg	4.4	Na, Mg
16	3kb2	2	2	unknown function	A6T8V8	338	2.20	0.195/0.262	41.61	Mg	4.1	Na, Mg
17	2gsl	2	3	unknown function	Q8RIL0	734 (6)	2.60	0.230/0.292	97.71	Mg	3.9	none
18	3c37	2	9	putative Zn-dependent peptidase Q74D82	Q74D82	445 (2)	1.70	0.219/0.254	58.16	Zn	-3.3	Na
19	2o18	2	11	Thiamine biosynthesis lipoprotein apbE	P0AB85	1226 (4)	2.20	0.233/0.284	152.83	Ca	2.5	Li
20	2gta	2	6	putative pyrophosphatase	P42979	377 (4)	2.90	0.225/0.263	57.47	Na	2.2	Na
21	2rjb	2	11	unknown function	A0A0H2UZX2	1572 (4)	2.60	0.233/0.281	211.12	Zn	2.0	none
22	4fj4[1]	2	1	unknown function	Q9HRE7	356 (2)	2.10	0.199/0.256	43.67	Na, Hg	-2.0	Na, K
23	3c5m	2	8	oligogalacturonate lyase (VPA0088)	Q87K10	1130 (3)	2.60	0.234/0.277	137.23	Mn	1.8	none
23	4jc0	2	7	Thermotoga maritima holo RimO	TM_1862	845 (2)	3.30	0.245/0.296	102.45	Fe	N/A	Na
25	2r7d	2	10	ribonuclease II family protein	Q9RYD0	1379 (3)	1.80	0.221/0.243	156.68	Mg	1.6	Mg
26	3cew	2	3	cupin protein (BF4112)	A0A380YRY7	472 (4)	2.31	0.217/0.246	55.32	Zn	-1.2	Na, Mg
27	2nvv[1]	2	13	Putative Acetyl-CoA hydrolase/transferase	Q7MVN7	2976 (6)	2.70	0.287/0.290	338.94	Zn	N/A	Ca
28	1ydn[1]	2	1	HMG-CoA Lyase	Q8YEF2	1132 (4)	2.30	0.271/0.304	126.71	Ca	N/A	Ca

The proteins are numbered in order of the magnitude of the highest *RSZD* and within that scoring system, from high to low molecular weight. The OM PDB ID and number of SeMet residues (MSE) are given per chain. A description of the protein, uniprot ID, the total number of residues per chain (with number of chains in parenthesis), resolution of the OM, and the *R* and R_{free} extracted directly from the PDB deposition, followed by the molecular weight of all the non-water atoms in the full sequence of the deposited model, are given. The metal or metals in the OM are listed, with the top *RSZD* by magnitude, and also any metals present in the crystallization conditions. Where no metal was identified in the OM, or experimental data were unavailable, the *RSZD* is given as N/A. 1. Proteins **3sm3**, **4fj4**, **1ydn**[24] and **2gsl** were samples without SeMet and so had no Se signal. In these cases, the number of non-substituted methionine residues is specified, instead of the number of SeMet residues.

Table S2 shows the SSGCID samples studied by both PIXE and XRFS. Each entry provides the PDB model ID, a description of the protein, the Uniprot ID, the number of residues, molecular weight, metal in the OM, the highest Alchemy derived *RSZD* for any metal in the model, and the presence of any metal in the crystallization conditions used to grow the crystal from which the model was obtained. The proteins are ordered according to the magnitude of the largest *RSZD* and then by their molecular weight from high to low if their *RSZD* values are identical. The metals present in the optimized conditions are reported in Tables 1 and 2. Run number is the experimental run described in the main text and S is the number of residues with Sulfur in the protein

Table S2. SSGCID samples studied by PIXE and XRFS.

	PDB ID	S	Run	Description	Uniprot ID	Residues	d_{max} (Å)	R/R_{free}	MW (kDa)	Metal in PDB	RSZD	Crystallization metal/s
1	4yet	8	3	Superoxide dismutase	O15904	398 (2)	1.75	0.143/0.176	45.92	Fe	99.9	Fe
2	4o5o	13	4	3-hydroxyacyl-CoA dehydrogenase	A0A0H3G6B0	518 (2)	1.40	0.136/0.169	54.86	Zn	-24.3	None
3	3uw2	11	3	Phosphoglucomutase/phosphomannomutase family protein	Q2SYG4	458	1.95	0.177/0.220	53.09	Zn	-18.3	None
4	5b8f	4	4	2-C-methyl-D-erythritol 2,4-cyclodiphosphate synthase	P57708	481(3)	1.45	0.138/0.169	54.31	Mg	17.6	Na
5	4wok	10	3	UDP-glucose 4-epimerase	A0A0H3ASR6	325	1.85	0.167/0.199	37.08	Zn	-17.4	None
6	5uf2	7	4	Ribose-5-phosphate isomerase A	B4RL16	223	1.40	0.135/0.164	25.83	Na	16.7	None
7	4dz4	9	4	Agmatinase	Q2T3W4	1900 (6)	1.70	0.152/0.175	214.04	Mn	-15.1	Na
8	4pcl	15	4	O-methyltransferase family protein	Q2GKC7	426 (2)	1.85	0.170/0.208	51.70	Mn	-12.5	None
9	4hdt	11	3	3-hydroxyisobutyryl-CoA hydrolase	G7CC99	340	1.60	0.160/0.189	37.95	Zn	-12.4	Zn
10	4u7x	14	4	Ribokinase:Carbohydrate kinase, PfkB	Q2YNY7	313	2.10	0.215/0.252	34.19	Na	12.1	Na

11	3r1j	6	3	Alpha-ketoglutarate-dependent taurine dioxygenase	A0A0H2ZSG9	487 (2)	2.05	0.171/0.200	67.77	Fe	-11.9	Na
12	4wjb	18	3	putative amidohydrolase/peptidase	B4EHA1	1628 (4)	1.95	0.173/0.213	183.8	Zn	-11.6	None
13	4y0e	3	3	Putative dioxygenase	B1MIR1	2414 (8)	1.90	0.160/0.195	304.58	Fe	-11.3	Mg, Ca
14	6ao1	17	4	Beta-lactamase domain protein	B2JKI6	1412 (4)	1.80	0.164/0.201	162.75	Ca	10.8	Ca
15	5umh	7	3	catechol 1,2-dioxygenase	A0A0H3KXJ8	617 (2)	1.35	0.130/0.163	70.72	Zn, Ca	-10.8	Fe, Zn, Ca
16	4f0l	18	3	Amidohydrolase from Brucella melitensis	BAB2_0307	897 (2)	2.05	0.148/0.184	99.13	Fe	-10.4	Na
17	3s6l	2	4	Hep_Hag family	Q3JFX2	777 (5)	2.30	0.175/0.207	89.78	Zn	10.1	Na,Zn
18	5u8o	17	3	Zn-dependent hydrolase	A0A0H3KRR5	1415 (4)	2.40	0.163/0.222	163.34	Zn, Ca	-9.1	Ca, Zn
19	5unb	11	4	Putative deoxyribonuclease-2	Q2T8B0	681 (2)	1.75	0.148/0.180	79.86	Cu	-8.2	Cu
20	4di0	3	3	Rubrerythrin	Q3JK21	279 (2)	1.90	0.169/0.200	31.93	Fe	-8.1	Fe
21	4o5f	10	4	Type III pantothenate kinase	Q2T1M2	506 (2)	1.55	0.156/0.183	60.72	K	-5.8	K, Mg
22	3mx6	10	3	Methionine aminopeptidase	Q9ZCD3	517 (2)	1.70	0.168/0.207	58.76	Mn	-5.7	Na
23	6n7l	19	4	Alcohol dehydrogenase	A0A077EGR8	4102 (12)	2.10	0.149/0.191	468.15	Zn	-5.3	Na
24	6d6j	6	4	HIT family hydrolase	Q5ZRW1	225 (2)	1.35	0.126/0.140	27.51	Zn	5.3	Na
25	3md7	15	4	Beta-lactamase-like	Q2YQ74	270	1.27	0.107/0.134	33.58	Mn, K, Na	5.1	K, Na
26	4j5i	1	3	Alpha-ketoglutarate-dependent taurine dioxygenase	A0QZ23	2056 (8)	2.60	0.211/0.234	268.88	Fe, Mg	4.6	Mg
27	3oce	17	4	Fumarate lyase:Delta crystallin	Q2YLW1	1842 (4)	2.58	0.196/0.245	204.8	Co	-4.0	Co
28	3u0a	8	4	Acyl-CoA thioesterase II TesB2	B2HN47	508 (2)	2.50	0.238/0.290	63.91	Na	3.8	None
29	5umf	13	3	Ribulose-phosphate 3-epimerase	B4RLV9	670 (3)	1.40	0.148/0.165	77.11	Zn	-2.5	Zn
30	4gri	14	4	Glutamate--tRNA ligase	O51345	963 (2)	2.60	0.223/0.285	118.93	Zn	2.4	K
31	6n1f	91	3	Oxidoreductase, 2OG-Fe(II) oxygenase family	Q3JFV4	842 (4)	2.05	0.208/0.242	108.52	Fe, Se	-2.4	Na
32	3o0m	6	4	HIT family protein	A0R291	274 (2)	1.90	0.157/0.191	33.28	Zn	-2.4	Ca,Na
33	3u0f	23	4	Beta-ketoacyl synthase	Q2YQQ9	407	1.25	0.122/0.134	43.97	Na	-2.2	Na
34	3r1i	16	3	Short-chain type dehydrogenase/reductase	B2HE09	501 (2)	1.85	0.164/0.197	58.33	Mg	2.1	Mg
35	3omf	4	4	Putative histidine triad family protein	C4LYI2	111	1.80	0.146/0.191	13.43	Zn	1.8	None
36	3r6f	5	3	HIT family protein	Q8SRE4	130	1.85	0.207/0.243	16.04	Zn	1.6	Zn
37	6caz	10	3	Peptide deformylase	Q5ZSC4	170	1.50	0.166/0.193	20.58	Ni	1.3	None
38	3r4t	19	3	4-aminobutyrate aminotransferase GabT	B2HN70	444	2.50	0.151/0.191	50.04	Na	1.0	None
39	5j46	12	3	Peptide deformylase 1	A0A0H3KB98	169	1.95	0.173/0.207	21.97	Zn	0.9	Zn
40	3oca	11	3	Peptide deformylase	Q2GI30	358 (2)	2.40	0.192/0.246	47.97	Zn	0.8	Zn
41	5kob	11	3	Peptide deformylase	Q13XB1	659 (4)	1.60	0.165/0.192	85.29	none	N/A	Mg
42	5i3e	11	3	Putative deoxyribonuclease-2	Q2T8B0	694 (2)	1.65	0.154/0.187	79.67	none	N/A	Cu

1. In the S (number of sulfur containing residues) column, **6n1f** is listed with its number of SeMet, not its sulfur residues. The proteins are numbered in order of the magnitude of the highest *RSZD* and then by molecular weight (MW) from high to low. The OM PDB ID and number of S atoms are given per chain. Tabulated are a description of the protein, uniprot ID, the total number of residues per chain (with number of chains in parenthesis), resolution of the OM, and the *R* and R_{free} extracted directly from the PDB deposition, followed by the MW of all the non-water atoms in the full sequence of the deposited model. The metal or metals in the OM are listed, with the top *RSZD* by magnitude and any metals present in the crystallization conditions identified. Where no metal was identified in the OM, or experimental data were unavailable, the *RSZD* is given as N/A.

Results

Supplementary tables S3 and S4 show the summary of the OM, and structural re-refinement outcomes from the NESG and SSGCID proteins. Any modelling and refinement over and above the simple replacement of the metal and subsequent PDB-REDO run is detailed in the notes section.

Table S3. Summary of structural outcome for the NESG proteins described in detail in the supporting information

NESG class I proteins – PDB original model inconsistent with PIXE data										
	Original			Re-refinement			Analysis			Notes
PDB ID	Metal	R and R_{free}	RSZD	Metal	R and R_{free}	RSZD	Delta R and R_{free}	Delta |RSZD|	Refined metal present in conditions[1]	
3bv9	Mg	0.212, 0.243	16.2	K	0.174, 0.220	3.0	0.038, 0.023	13.2	No	With the correct metal modeled a ligand became visible and this was also modeled.

4py9	Na	0.199, 0.239	12.4	Mn	0.197, 0.234	2.2	0.012, 0.005	10.2	No	For Na and Mn, the occupancy was refined with REFMAC alone before PDB-REDO. With Mn modeled, K and Ca ions were identified and added to the appropriate density.
3mel	Mg	0.199, 0.238	10.8	Fe	0.168, 0.249	2.1	0.0316, -0.011	8.7	No	Not that the RSZD of 10.8 came from an Mg ion that was identified as an SO4 ion. The new RSZD is for an Fe ion replacing another Mg ion that had an RSZD of 6.2, a delta RSZD of 4.6
2f6k	Mn	0.168, 0.199	9.4	Fe	0.166, 0.198	0.7	0.002, 0.001	8.7	No	
2gzx	Ni	0.176, 0.209	7.4	Fe	0.171, 0.206	1.5	0.005, 0.003	5.9	No	
3bb6	Zn	0.194, 0.220	6.9	Fe	0.190, 0.209	4.3	0.004, 0.011	2.6	No	Water coordinated with two of the Fe ions could clearly be modeled.
3guw	Zn	0.243, 0.321	6.7	Cu	0.242, 0.321	2.0	0.001, 0.000	4.7	No	A third Cu was modeled into a tri-nuclear site supported by the difference electron density.
2h6l	Zn	0.144, 0.180	-6.4	Co	0.144, 0.179	3.2	0.000, 0.001	10.3	No	
2bdr	Na	0.128, 0.161	8.6	Na	0.135, 0.163	-4.9	-0.007, -0.002	13.5	Yes	One of the Na ions appeared misplaced and while the RSZD decreased, the overall R and R_{free} increased with this modeled. Na was undetectable by PIXE under the experimental setup used but because of the large RSZD, the OM was classed as inconsistent with the experimental data.
2gwg	Zn	0.160,0.183	-5.1	Cu	0.152, 0.187	-3.6	0.008, -0.004	1.5	No	With the Cu ion modeled, acetate ions we seen next to the Cu ions.
2gsl	Mg	0.195, 0.238	3.9	N/A	N/A	N/A	N/A	N/A	No	
4jc0	Fe	0.252, 0.327	2.6	N/A	N/A	N/A	N/A	N/A	Yes	A metal cluster was in the crystallization conditions and there was no evidence for any other metal despite the positive electron difference density seen. The RSZD is likely due to the highly electrophilic nature of the cluster.
2gta	Na	0.210, 0.250	2.2	N/A	N/A	N/A	N/A	N/A	Yes	
2rjb	Zn	0.184, 0.235	-5.9	Fe	0.184, 0.234	3.7	0.000, 0.001	9.6	No	
4fj4	Na	0.200, 0.235	-2.0	N/A	N/A	N/A	N/A	N/A	Yes	
3c5m	Mn	0.206, 0.246	1.8	Ni	0.198, 0.243	1.6	0.008, -0.003	0.2	No	
3cew	Zn	0.180, 0.227	-1.2	Zn	0.174, 0.222	-1.2	0.006, 0.005	0.0	No	
1ydn	Ca	N/A	N/A	N/A	N/A	N/A	N/A	N/A	Yes	
NESG class II proteins – extra metals indicated experimentally										
	Original			Re-refinement			Analysis			Notes
PDB ID	Metal	R and R_{free}	RSZD	Metal	R and R_{free}	RSZD	Delta R and R_{free}	Delta |RSZD|	Refined metal present in conditions?	
3sm3	Ca	0.191, 0.226	8.5	Cu	0.189, 0.233	2.2	0.002, -0.007	6.3	No	The Cu signal was significantly higher than others during the PIXE data collection.
2hf1	Zn	0.210, 0.234	-3.4	Zn	N/A	N/A	N/A	N/A	No	Although PIXE data indicate Ca, Se, and Co in addition to the Zn, the OM appears correct.
2h28	Mg	0.188, 0.211	-5.9	Ca	0.187, 0.215	-3.9	0.001, -0.004	2.0	No	Anomalous data or complementary techniques would be required to unambiguously differentiate between Mg and Ca.
4h9d	Zn, Mg	0.205, 0.276	4.4	Co	0.196, 0.262	2.0	0.009, 0.014	2.4	Mg only	Modeling Co and waters coordinated to the Mg ions resulted in a slight improvement over Zn.
2o18	Ca	0.215, 0.254	2.5	K	0.208, 0.254	2.7	0.007, 0.000	0.2	Yes	
2nvv	Zn	0.264, 0.321	1.1	Fe	0.257, 0.317	1.1	0.007, 0.004	0.0	No	There was insufficient X-ray data for the re-refinement with more recent software.
NESG class III proteins – PIXE data agree with the model										
	Original			Re-refinement			Analysis			Notes
PDB ID	Metal	R and R_{free}	RSZD	Metal	R and R_{free}	RSZD	Delta R and R_{free}	Delta |RSZD|	Refined metal present in conditions?	
3goc	Mg	0.156, 0.185	-12.2	Mg	0.121, 0.162	-1.0	0.035, 0.023	11.2	Yes	Mg was undetectable by PIXE under the experimental setup used but because of the large RSZD, the Mg occupancy was refined with REFMAC alone before PDB-REDO. Repositioning of some water molecules was necessary.
3c37	Zn	0.200, 0.229	5.7	Zn	0.200, 0.229	-3.1	0.000, 0.000	8.8	No	The metal was not changed or moved but residue Glu 162, close to the ion was repositioned.
3kb2	Mg	0.202, 0.257	4.1	N/A	N/A	N/A	N/A	N/A	Yes	

Table S4. Summary of structural outcomes for the SSGCID proteins described in detail in the supporting information

SSGCID class I: PDB original model inconsistent with PIXE data and/or XRFS data										
	Original			Re-refinement			Analysis			Notes
PDB ID	Metal	R and R_{free}	RSZD	Metal	R and R_{free}	RSZD	Delta R and R_{free}	Delta |RSZD|	Refined metal present in conditions ?	
5b8f	Mg	0.154, 0.174	17.6	Fe, Mn, Ni, Zn	0.144, 0.163	-16.7	0.010, 0.011	0.9	No	The identity of the metal was ambiguous and the values for Fe are given. Other methods would be needed to model the metal site accurately as described in the supplementary material.
5uf2	Na	0.119, 0.148	16.7	Zn/Ni	0.121, 0.150	2.1	-0.002, -0.002	14.6	No	An equal mixture of Ni and Zn at reduced occupancy are present. PDB-REDO failed on this so REFMAC5 was used refining occupancy and with recommended parameters. Ni and Zn were refined in one site at 28% each, and Zn in the other at 36% occupancy.
4pcl	Mn	0.171, 0.197	-12.5	Ni	0.142, 0.192	-3.5	0.029, 0.005	9.0	No	Ni was determined to be present.
4hdt	Zn	0.157, 0.189	-13.9	Zn	0.158, 0.187	-3.6	-0.001, 0.002	10.3	Yes	Zn was not detected with the PIXE or XRFS data but is present in the crystallization conditions. The Zn sites had alternate positions.
4u7x	Na	0.197, 0.232	12.1	Ni	0.193, 0.227	2.5	0.004, 0.005	9.6	No	One Na was replaced with Ni and it appears at less than unit occupancy.
4y0e	Fe	0.177, 0.202	-11.3	Ni (Zn)	0.156, 0.207	2.3	0.021, -0.005	9.0	No	The Ni occupancy was refined with REFMAC5. PDB-EDO failed due to a tortoize error (a known issue at the time) and the refinement continued with parameters recommended by REFMAC5. The Ni occupancy ranged from 77 to 89%.
6ao1	Ca	0.178, 0.201	10.8	Fe, Zn, Mn, Ni, Cu, Se	0.171, 0.217	-2.1	0.007, -0.016	8.7	No	The metal was re-modeled as a mixture of Fe and Zn based on the XRFS data signal ratio. The refined occupancy was ~50% for each but with one site containing 74% Fe and 26% Zn.
5unb	Cu	0.183, 0.209	-8.2	Cu	0.154, 0.181	2.1	0.029, 0.028	6.1	No	Cu was present in the crystallization conditions.
4o5f	K	0.165, 0.189	-4.4	Ni (Se)	N/A	N/A	N/A	N/A	N/A	
3mx6	Mn, Na	0.170, 0.203	-5.7	Fe (Mn)	0.160, 0.199	0.0	0.010, 0.004	5.7	No	Described in detail above.
3oce	Co	0.190, 0.232	-4.0	Zn	0.188, 0.236	3.4	0.002, -0.004	0.6	No	The crystallization conditions contained Co, and Zn was detected experimentally in the sample. The metal site is likely a mixture.
3u0a	Na	0.183, 0.232	3.8	none	0.183, 0.232	N/A	0.000, 0.000	N/A	N/A	No metal was present in the new model.
6n1f	Fe	0.221, 0.252	-2.4	none	0.221,0.252		0.000, 0.000		No	Fe in the OM was at 90% occupancy, the Ni was modeled at 83%.
3u0f	Na	0.122, 0.134	-2.2	Ca, K (Ni)	N/A	N/A	N/A	N/A	N/A	
3r1i	Mg	0.164, 0.197	2.1	Ca, Ni, Fe	N/A	N/A	N/A	N/A	N/A	
3r4t	Na	0.151, 0.191	1.0	Ca	N/A	N/A	N/A	N/A	N/A	
5i3e	none	0.154, 0.187	N/A	Ni, Ca (Cu)	N/A	N/A	N/A	N/A	N/A	This was a control sample with no metal expected. Despite a metal signal, no evidence was detected for an ordered metal in the OM electron density map.
SSGCID class II proteins – extra metals indicated experimentally										
	Original			Re-refinement			Analysis			Notes
PDB ID	Metal	R and R_{free}	RSZD	Metal	R and R_{free}	RSZD	Delta R and R_{free}	Delta |RSZD|	Refined metal present in conditions?	
4o5o	Zn	0.157, 0.178	-24.3	Ni	0.155, 0.176	0.7	0.002, 0.001	23.6	No	Ni was modeled at a reduced occupancy.
3uw2	Zn	0.177, 0.215	-18.3	Mg	0.176, 0.213	-0.9	0.001, 0.002	17.4	No	
4wok	Zn	0.167, 0.193	-15.1	Zn	0.162, 0.196	2.6	0.004, -0.003	13.5	No	Zn was modeled at reduced occupancy (60%) and an alternate chain was removed. Further modeling was needed.
4dz4	Mn	0.156, 0.180	-13.7	Fe/Mn	0.131, 0.158	3.1	0.025, 0.022	10.6	No	Several residue shifts were modeled, additional waters were located, and potential ligands were seen but not modeled. The Fe occupancy ranged from 50 to 86%.
3r1j	Fe	0.173, 0.198	-11.9	Fe	0.169, 0.190	2.2	0.004, 0.008	9.7	No	The Ni was modeled at a reduced occupancy.
4wjb	Zn	0.173, 0.216	-14.7	Zn	0.172, 0.217	1.4	0.001, 0.001	13.3	No	The OM had partial occupancy for Zn and the experimental model looks to have a Fe/Zn mixture.

5umh	Zn, Ca	0.138, 0.169	-10.8	Zn, Ca	0.132, 0.164	-4.9	0.006, 0.005	5.9	Yes	The OM had a reduced occupancy for Zn, the replacement Fe is at 100% occupancy. Occupancy was not refined for this.
4f0l	Fe	0.152, 0.180	-10.4	Fe	0.135, 0.185	-0.7	0.017, -0.005	9.7	No	
3s6l	Zn	0.171, 0.208	8.4	Zn	0.170, 0.209	2.5	0.001, -0.001	5.9	Yes	
5u8o	Zn, Ca	0.178, 0.219	-9.1	Zn, Ca	0.178, 0.219	-3.2	0.000, 0.000	5.9	Yes	Three metals were tested in the Zn site with the R and R_{free} staying identical but the RSZD reducing to -3.6 for Fe and -3.2 for Mn. Metrics for Fe are given based on the XRFS data.
4di0	Fe	0.170, 0.199	-8.1	Fe	0.149, 0.191	-4.3	0.021, 0.009	5.6	No	Fe was modeled at reduced occupancy.
6n7l	Zn	0.162, 0.209	-6.2	Zn	0.158, 0.188	-3.8	0.004, 0.021	2.4	No	
6d6j	Zn	0.110, 0.124	5.3	Zn	N/A	N/A	N/A	N/A	No	
3md7	Mn, K, Na	0.129, 0.147	5.1	Zn	N/A	N/A	N/A	N/A	N/A	Mn was confirmed independently.
4j5i	Fe, Mg	0.197, 0.230	4.6	Mn, K, Na	0.197, 0.230	2.1	0.000, 0.000	2.5	No	
5kob	Fe	0.165, 0.192	-6.2	Zn	0.147, 0.185	2.5	0.018, 0.007	3.7	No	It is likely that the metal sites are a mixture of Zn, Fe and possibly Ni.
4yet	Fe	0.151, 0.173	3.3	Fe, Mn	0.125, 0.168	0.5	0.026, 0.005	2.8	No	A mixture of Fe (75%) and Mn (25%) were modeled based on the XRFS data.
5umf	Zn	0.130, 0.151	-2.5	Zn	0.122, 0.148	-2.7	0.008, 0.003	-0.2	Zn only	
4gri	Zn	0.223, 0.285	2.4	Zn	N/A	N/A	N/A	N/A	No	
3o0m	Zn	0.157, 0.191	-2.4	Zn	N/A	N/A	N/A	N/A	No	
3omf	Zn	0.146, 0.191	1.8	Zn	N/A	N/A	N/A	N/A	No	.
3r6f	Zn	0.207, 0.243	1.6	Zn	N/A	N/A	N/A	N/A	Yes	
6caz	Ni	0.189, 0.214	-4.9	Ni	0.173, 0.198	-3.1	0.016, 0.016	1.8	No	
3oca	Zn	0.192, 0.246	0.8	Zn	N/A	N/A	N/A	N/A	Yes	
5j46	Zn	0.173, 0.207	0.9	Zn	N/A	N/A	N/A	N/A	Yes	

In this Supporting Information, we present the results of metal identification analyses using PIXE and XRFS for the 62 samples not described in detail in the main paper.

NESG Class I proteins – PDB original model inconsistent with PIXE data

For the class I NESG samples, **3kb2, 2gsl, 2gta, 4fj4, 2r7d**,[1] and **1ydn**,[2] the OMs, while not having the modeled metals detected by PIXE and/or XRFS, they were consistent with metals that were present in the crystallization conditions, or had metals undetectable by the PIXE or XRFS protocols used (Mg, Na), so they were not re-refined. The OMs for **3kb2** and **2gsl** have *RSZD*s of 4.1 and 3.9, respectively, both explained by positive density for a coordinated but unmodeled water close to a metal ion.

PDB ID **3vb9** is a structural model of VPA0735 from *Vibrio parahaemolyticus*. This protein has four chains and is modeled with two Mg ions in two chains, one in each having significant excess electron density, one Mg ion in the third chain, and two in the fourth chain, again one of these having significant unexplained electron density. The PIXE data indicate the presence of K, but under the experimental arrangement used, is insensitive to Mg. Mg is present in the crystallization conditions. In the three chains with excess electron density, K ions were modeled in place of the Mg ions. When this new model was crystallographically refined, the *R* and R_{free} were 0.211 and 0.243 compared to 0.212 and 0.244 for the OM. The reductions in *R* and R_{free} were insignificant, and the model with the K ions still had structured positive electron density around four of the ions. For these ions, the CMMS indicated clear issues with the OM model, flagging problems with the elemental composition of the coordination sphere, the metal-ligand distances, and geometry etc. These ions were removed, and the model re-refined. The resulting difference map indicated positive density that clearly fit the sulfonic acid functional group of another component in the crystallization conditions, (3-(N-morpholino)propanesulfonic acid) (MOPS). MOPS was inserted into the re-refined model. The resulting R and R_{free} decreased further, and the improved electron density indicated a register shift in one chain. Once corrected, the final R and R_{free} were significantly reduced to 0.188 and 0.233, The model was manually inspected, a small number of misidentified amino acid residues corrected, and the occupancy of and MOPS refined with REFMAC5. The final *R* and R_{free} were 0.174 and 0.223, with the MOPS clearly visible in the electron density. The CMMS indicated a far more acceptable fit with K rather than Mg. We conclude that the metal is K in half of the original sites, and the other metal site is the sulfonic acid functional group of MOPS. The model was redeposited in the PDB updating the OM.

PDB ID **4py9** is a structural model of an exopolyphosphatase-related protein from *Bacteroides Fragilis*. There is one chain in the OM with four Na ions, two with positive difference electron density, and two with negative difference density surrounding them. The CMMS was only able to evaluate basic information on the occupancy and *B*-factors of the sites. The PIXE data show the presence of Mn at > 2×LOD with K and Na present in the crystallization conditions. REFMAC5[3] was used outside the PDB-REDO pipeline to refine the Na and Mn occupancy and then these values used for subsequent re-refinement in PDB-REDO. For the positive difference density, Mn was initially modeled since the density supported a much heavier element, and the occupancy of both the Na and Mn refined, resulting in *R* and R_{free} pf 0.197 and 0.233, compared to the OM with 0.199 and 0.239. Other positive difference density became apparent in the Mn containing

model, and K, Na, and Ca ions could be added reducing the R further to 0.197 but increasing R_{free} to 0.234. The CMMS deemed the Na to be borderline to acceptable. The *RSZD* was 2.2, compared to the original of 12.4.

PDB ID **3goc** is a structural model of endonuclease V (SAV1684) from *Streptomyces avermitilis*. The model has two chains with a binuclear metal site containing two Mg ions in each chain. There was positive and negative difference density in the vicinity of the metal site and an *RSZD* of -12.2. The CMMS flagged a high percentage of unoccupied sites and the geometry was assessed as an outlier to borderline. The PIXE data detected no evidence for any metals in the protein at > 2×LOD but Na and Mg were present in the crystallization conditions. Endonuclease V is thought to have a catalytic and regulatory process related to this metal site with several metals, including Mg known to bind.[4] Re-modelling with a reduced occupancy for the Mg ions and repositioning the waters improved the model. In this case the RSMD rather than the PIXE data identified an issue and this was rectified, improving the *RSZD* to -1.0 with an R and R_{free} of 0.121 and 0.162 compared to the OM at 0.156 and 0.185 respectively, indicating a very significant improvement in the final model.

PDB ID **2f6k** is a structure of amidohydrorolase II from *Lactobacillus plantarum*. Amidohydrolase II, a member of the metallo-dependent hydrolase superfamily, typically utilizes various metal ions for its catalytic activity, including Zn and Mn and in some cases it may also bind Fe as a cofactor.[5] The OM has an Mn ion in both of the two chains present and the CMMS flags these as acceptable. The PIXE data show no Mn, but indicate K, and Fe at > 2×LOD. Replacing the Mn with Fe and re-refining results in R and R_{free} of 0.166 and 0.198 compared to the OM with 0.168 and 0.199. The *RSZD* reduced from 9.4 to 0.7 with Fe modeled, a further indication of an improved structure with Fe as the metal in both chains.

PDB ID **2gzx**, TatD deoxyribonuclease MW0446 from *Staphylococcus aureus* was modeled with a Ni ion. K and Fe were observed in the PIXE data although there was no Ni seen. Deoxyribonuclease requires Ca or Mg to function[6] and Mg was in the crystallization condition. A positive *RSZD* indicates that the electron density is underestimated. There are two chains, and the Ni ions are in a binuclear site in both chains with positive density associated with both. consistent with a water molecule in between them. Removing the Ni ions and replacing them with Fe ions and the indicated water, with re-refinement, resulted in R and R_{free} of 0.171 and 0.206 for the Fe ion, a slight reduction compared with the 0.176 and 0.209 for the OM, and an *RSZD* of 1.5 compared to the original of 7.4. There was no noticeable difference density in the new model, indicating that Fe is most probably the correct metal.

PDB ID **3bb6** is a structural model of the P64488 protein from *E.coli* (strain K12), a protein of unknown function. This has four chains and a Zn ion is modeled in each, coordinated with two His residues and an Asp. Two of these sites have significant positive electron density associated with them. No Zn is detected in the PIXE data, but K is seen and Fe at > 2×LOD. The crystallization condition contained Na, but no Zn. Replacing the Zn ions with Fe and re-refining gave R and R_{free} of 0.190 and 0.209 compared to the original of 0.194 and 0.220. Water coordinated with two of the Fe ions could clearly be modeled. The *RSZD* was 4.3 compared to the OM with 6.9. This was consistent with the CMMS results where the *B*-factor, metal-ligand and geometry were all flagged as outliers and Fe was identified as one of the potential metals in the site. Thus all the available evidence points to Fe being the correct assignments.

PDB ID **2h6l** is a structural model of a metal-containing protein from *Archaeoglobus fulgidus*. The OM consists of three chains, each with a Zn ion coordinated with three His residues and an acetic acid molecule. Two of the three ions have no difference density associated with them, but one has negative difference density. However, PIXE data do not indicate the presence of Zn > 2×LOD, but do show K, Ca and Se, as well as Fe and Co at the > 2×LOD level. The R and R_{free} of the OM were 0.144 and 0.180 respectively. There are no biochemical data to indicate which metal, Fe or Co, is more likely, and the model was re-refined with both Fe and Co replacing the Zn ions. For Fe, the R and R_{free} were 0.145 and 0.179, and for Co, 0.144 and 0.179 respectively. Both the Fe and Co are compatible with the difference maps with some excess positive difference density seen for the Fe case at two of the sites. The *RSZD* for Fe was 4.6 and that for Co was 3.6 as compared to the original *RSZD* of -6.4. In this case, Co is a better fit to the diffraction data. The CMMS flagged the outlier metal-ligand distances and proposed a number of metals including Co, so most probably Co is the correct metal.

PDB ID **2bdr** is a structural model of a putative ureidoglycolate hydrolase from *Pseudomonas putida*. Ureidoglycolate hydrolase is a metalloenzyme, and its activity involves the coordination of metal ions, primarily Mn or Zn. These metal ions play a catalytic role in the hydrolysis of ureidoglycolate. There are two chains modeled with three Na ions in one chain. In the first chain one Na ion has unmodeled electron density close to it, the second shows no excess density, and the third has excess positive difference density nearby. The PIXE data indicate K at >3×LOD and also Mn and Fe at >2×LOD. Na is present in the crystallization conditions. One of the Na ions appeared to be misplaced as the ion was linking two chains. This was corrected and the model re-refined. The resulting R and R_{free} were 0.135 and 0.163 compared with 0.128 and 0.161 for the OM but the

RSZD was improved from 8.6 to -4.9. In this case there is a clear improvement with the difference density, for the metal site, but also features that would benefit from further modeling and refinement as well as having anomalous diffraction data in order to definitively assign the correct metal ions.

PDB ID **2rjb** is an uncharacterized protein, YdcJ (SF1787), from *Shigella flexneri*. Four chains are modeled with one Zn ion in each chain. PIXE does not detect the presence of Zn, but K and Fe are indicated at > 3×LOD, and Ca at > 2×LOD. Cu is also indicated at 3× the average signal of Cu that was observed in all of run 2 PIXE spectra. Calibrating to the known Se content suggests 3 Fe ions. Modeling Fe into the Zn sites and re-refining gave R and R_{free} values of 0.184 and 0.234 respectively; the OM had a slightly higher R_{free} of 0.235. The Fe ions reproduce the electron density well, and minimal difference density is seen except for a couple of clear areas indicating water molecules coordinated with the Fe, the two His residues, and the Glu in the metal site. These are also present in the OM, and it seems likely that the *RSZD* peak stems from them. The new *RSZD* was 3.7 compared to the original of -5.9. With Cu ions replacing the OM zincs, the R and R_{free} were 0.184 and 0.232 and the *B*-factors were much larger than for the surrounding atoms. Thus Fe seems to be the correct metal ion in this case.

PDB ID **3c5m** is a structural model of oligogalacturonate lyase (VPA0088) from *Vibrio parahaemolyticus*. The OM contains three chains with an Mn ion modeled in each, coordinated to three His and one Gln residue. The PIXE data show Ni at >2×LOD but indicate no Mn, and Mn is not in the crystallization conditions. There has been a suggestion that pectate lyases could harness novel cofactors including Ni.[9] Replacing the Mn ions with Ni and refining resulted in R and R_{free} of 0.198 and 0.243 compared to 0.206 and 0.246 for the OM. In this case, we conclude that the metal in the structural model should be Ni, presumably incorporated from the purification process. The original *RSZD* was 1.8 and for the Ni was 1.6, a small improvement but consistent with the PIXE results.

PDB ID **3cew** is a model for a cupin protein from *Bacteroides fragilis*. The OM has four chains with a Zn ion in each. PIXE data indicate the presence of Ca, Fe, and Se, and Na and Mg are present in the crystallization conditions. The OM gives R and R_{free} of 0.180 and 0.227 when re-refined. The *B*-factor for the Zn is somewhat higher than the residues to which it is attached. Replacing the Zn ions with Fe and re-refining gave and R and R_{free} of 0.174 and 0.222. The re-refined model and electron density was completely consistent with this being an Fe binding protein. In most cuprin domain containing proteins the metal is Fe.[10] The *RSZD* for Fe was -1.2, the same as for the OM.

NESG class II proteins – extra metals indicated experimentally

PDB ID **3sm3** is a structural model of a SAM-dependent methyltransferase Q8PUK2_METMA from *Methanosarcina mazei*. It has one chain containing two Ca ions, one of which is partially occupied with no difference density, and the other fully occupied with significant positive difference density. The PIXE data indicate K and Ca. The partially occupied Ca ion was remodeled as a K ion, and a water molecule was added into the coordination sphere around the fully occupied Ca ion. The R and R_{free} for the new model were 0.186 and 0.229, compared to 0.19.1 and 0.226 for the original. The K ion still showed significant positive electron difference density, but the water added did fit the experimental data. The PIXE data had a Cu contamination signal, but the level of Cu signal in this sample was significantly above the average for the other samples. Modelling Cu into the metal site resulted in R and R_{free} of 0.189 and 0.233 with an *RSZD* of 2.2 compared to the original of 8.5. While the R_{free} is higher, based on the experimental evidence, it is likely that the metal is Cu but at less than unity occupancy due to the elevated *B*-factors observed. The CMMS flagged the OM as a problem, and suggested Cu as one of the likely metals for the Ca ion in the OM with positive difference density. No further re-refinement took place.

PDB ID **2hf1** is a structural model of the putative tetraacyldisaccharide-1-P 4-kinase from *Chromobacterium violaceum*. The OM has two chains with a Zn ion in each. The PIXE data indicate Ca, Se, Zn, and Co at greater than 3× LOD with normalized signals of 0.49, 0.22, 0.18 and 0.11. Na is present in the crystallization conditions. There is positive difference electron density at one of the Zn ion positions, the other having no significant difference density, but a dual conformation for an Asp residue. Given the presence detected by PIXE, the identification of Zn in the first site appears accurate. The second site displaying positive difference density was refined as a Se ion giving an R and R_{free} of 0.208 and 0.244 and an *RSZD* of 4.4, the positive density potentially indicating electrons in a bond. The residues surrounding the Se are three Cys and an Asp, compatible with the Se but the OM had an R and R_{free} of 0.210 and 0.234, and an *RSZD* of -3.4. In this case, Zn appears to be the correct assignment, with the Se signal probably arising from the SeMet incorporation. In the OM, there is positive electron density modeled as water molecules that supports the presence of imidazole, but this was not modelled and no further re-refinement was carried out. The CMMS regarded the OM as acceptable but with border-line metal-ligand distances.

PDB ID **2h28** is a structural model of YeeU from *E. coli*.[7] YeeU is part of the toxin-antiotoxin system controlling cell growth and response to stress conditions.[8] The OM has a Mg

ion in each of two chains with the PIXE data indicating Fe and Ca (at >2×LOD) and there is Mg in the crystallization conditions. There is both positive and negative difference density around the Mg ion in the first chain, with a peak *RSZD* of -5.9. Fe was substituted into the OM. The *R* and R_{free} for refinement for the OM and Fe substituted models were 0.188 and 0.211, and 0.190 and 0.211 with an *RSZD* of 6.6 respectively. The electron density did not support the presence of Fe. Re-modelling Ca into the sites gave an *R* and R_{free} of 0.187 and 0.215 with an *RSZD* of -3.9. The CMMS flags the Mg ions as outliers but without further experimental data it is difficult to evaluate which is more likely to be present, Mg or Ca.

PDB ID **4h9d** is a structural model of Mn-dependent Gme HNH nicking endonuclease from *Geobacter metallireducens*. Three chains are modeled, with a Zn ion in two of those and an Mg in the other. There is positive difference density associated with one of the Zn ions. In the chain containing a Mg ion, the *RSZD* of 4.4 appears to be associated with positive difference density compatible with two coordinated waters. The PIXE data indicate the presence of Co, K, and Zn in ratios of 0.40, 0.30 and 0.26, and Na and Mg are present in the crystallization conditions. The OM had *R* and R_{free} of 0.201 and 0.270, and replacing them with Co resulted in *R* and R_{free} of 0.201 and 0.268. Modeling waters coordinated with the Mg reduced the *R* and R_{free} to 0.196 and 0.262 and the *RSZD* to 2.0. The CMMS suggested that the Zn sites were acceptable but flagged the Mg site. From the available data it is hard to draw a definitive conclusion in this case.

PDB ID **2o18** is a structural model of thiamine biosynthesis lipoprotein apbE from *E. coli*. In the four chains of the OM, a Ca ion is present in each. The Ca ions have significantly higher *B*-factors than the surrounding residues but Ca was identified in the PIXE data along with K. The Ca ions appear nicely modeled in the density. Replacing them with K and Li (present in the crystallization conditions) resulted in *R* and R_{free} of 0.208 and 0.254, and 0.209 and 0.254 respectively, compared to the 0.215 and 0.254 for the OM. The Li ions were clearly incorrect, showing significant excess positive electron difference density. The *RSZD* was 2.7 compared to the original of 2.5. There was no visible difference between the maps for the Ca and K ions, and given the PIXE data, the correct ion is K. This result was also supported by the CMMS that flagged metal-ligand distances and suggested K as the correct ion.

PDB ID **2nvv** is a structural model of a putative Acetyl-CoA hydrolase/transferase PG1013 from *Porphyromonas gingivalis*. The OM has six chains with a single Zn ion in each of the first five chains. The PIXE data indicate no Zn but show the presence of Ca and K, with Fe > 2×LOD. There is no difference electron density and the *RSZD* indicates the Zn ions fit the electron density well, but the *B*-factor of the ions are approximately three times that of the surrounding residues. PDB-REDO noted that there was insufficient completeness in the experimental X-ray data for the re-refinement to complete. Troubleshooting this indicated a possible error with the OM deposition and a molecular replacement was carried out against the data using a monomer from the OM and Phaser.[11] This was successful and the new model was refined against the deposited experimental data. The OM with Zn gave *R* of 0.264 and R_{free} of 0.321 and an *RSZD* of 1.1. The Zn ions were replaced with Fe, and the model re-refined yielding *R* and R_{free} of 0.257 and an RSZD of 1.1. The resolution of the data was 2.4Å and given the PIXE data, the *B*-factors of the Zn ions, and the *B*-factors for the Fe ions were examined. These were consistent with their neighbors. There is a correlation between occupancy and *B*-factor especially with limited observations. In this case, despite the large difference in atomic number and therefore scattering power, the indications from the PIXE data, the similarity between the Zn and Fe , and the difference in *B*-factor, all point to the correct metal being Fe (or potentially predominantly Fe , not Zn).

NESG class III proteins – PIXE data agrees with the model

Only one sample had PIXE data in complete agreement with the model, PDB ID **3c37**. This is a structural model of the putative Zn-dependent peptidase Q74D82 from *Geobacter sulfurreducens* PCA. Zn was detected at > 2×LOD in the PIXE data and Na was present in the crystallization conditions. The OM has two chains, both with a Zn ion, each showing no evidence of any difference density around the metal ion, but there was positive difference density associated with Glu 162. Repositioning Glu 162 gave *R* and R_{free} of 0.200 and 0.229, the same as the OM, with an *RSZD* of -3.1 compared to the OM with an *RSZD* of 5.7. No further re-refinement took place.

SSGCID class I proteins - PDB original model inconsistent with PIXE data and/or XRFS data

For the SSGCID class I samples, the XRFS, PIXE data, metals present in the crystallization conditions, and occupancy considerations were in agreement with the PDB models for IDs **4o5f**, **3u0f**,[12] and **3r1i**, [13] and no re-refinement of these was carried out. In addition, sample **5i3e**[14], used as a control as it had no metal modelled, had a PIXE signal for Ni and Ca and a trace indication of Ni in the XRFS data, but there was no evidence of an unoccupied metal ion site in the electron density and no re-refinement of the OM was carried out.

PDB ID **5b8f** is a 2-C-methyl-D-erythritol 2,4-cyclodiphosphate synthase from *Pseudomonas aeruginosa,* a gram-negative, aerobic, non-spore forming rod that is capable of causing a variety of infections in both immunocompetent and immunocompromised hosts.[15] The asymmetric unit consists of three identical chains with an Mg ion present in

each. One fits the electron density well, and the other two are associated with negative electron density in the F_o-F_c map, one significantly so. The XRFS data indicated Zn, Fe, Cu, Ni, and Mn at normalized ratios of 0.59, 0.17, 0.15, 0.06, and 0.03, respectively. The PIXE data are consistent with this, identifying Fe, Mn, Ni, and Zn. A study on the same protein from *Plasmodium falciparum* noted the requirement for divalent metal ions such as Mg as in the OM, or manganese for catalytic activity.[16] The negative electron density could be explained by either Mg or any of the metals detected at a reduced occupancy. The OM R and R_{free} were 0.154 and 0.174 respectively. There is significant unmodeled positive electron density in the map coordinated by Asp 8, His 10, and His 42 in the third chain. This is very similar to the zinc enzyme *Escherichia coli* 2-C-methyl-d-erythritol 2,4-cyclodiphosphate synthetase that contains a Zn and Mn ion acting in concert.[17] Removing the Mg ion showing significant negative difference density in chain C and replacing this largest peak with Fe, and then Zn, gave R and R_{free} of 0.144 and 0.163, and 0.145 and 0.163, with *RSZD*s of -16.7, and -24.2, respectively. The OM had an *RSZD* of 17.6. The CMMS flagged the two Mg ions in chain A and C as borderline outliers. In this case the XRFS and PIXE data could not unambiguously determine the metal identities and other methods would be needed to confirm them.

PDB ID **5uf2** is a ribose 5 phosphate isomerase A from *Neisseria gonorrhoeae,* a sexually transmitted bacterium causing gonorrhea. The difference electron density maps indicate that a larger atomic weight atom is present at the modeled Na position. The XRFS data show Zn and Ni, with ratios of 0.55 and 0.45. The PIXE data were in agreement and also indicated Mn. Modeling Zn and Ni into the single metal site and re-refining gave R and R_{free} of 0.120 and 0.154 with an *RSZD* of -31.7 for Zn, and 0.121 and 0.148 with an *RSZD* of -29.0 for Ni, compared to 0.119 and 0.148 for the OM. There is significant remaining negative difference density around both the Ni and Zn, suggesting that based on the XRFS and PIXE data, both may be present at reduced occupancy. REFMAC5[3] was used to refine the occupancy for both, allowing for a less than unity total, and the model was resubmitted to PDB-REDO. The Zn/Ni site had respective occupancies of 28% for each ion, and the Zn site 35% with an *RSZD* of 1.3 and R and R_{free} of 0.124 and 0.152 respectively. While the R and R_{free} are worse than the OM, the experimental data supports the Zn/Ni model. Anomalous X-ray diffraction studies or other techniques would be needed to determine a more accurate model.

PDB ID **4hdt** is the model of a Carnitinyl-CoA dehydratase from *Mycobacterium thermoresistibile.*[18] The *RSZD* for the OM containing five Zn ions is -13.9, with the XRFS data indicating Ca and Se at >2×LOD. The PIXE data showed Ca. No Zn was detected in the sample but both Zn and Na are present in the crystallization conditions. The difference electron density suggested an alternate Zn position for three of the ions with partial occupancy for each. REFMAC5[3] was used to refine the occupancy, ranging from 75% to 33%, and then these occupancies, ~50% for each, were used with PDB-REDO to re-refine the model. The original R and R_{free} were 0.166 and 0.200 and those of the re-refined model were 0.158 and 0.187 with an *RSZD* of -3.6. The CMMS for the OM indicates that three of the Zn ions are acceptable, with two having outliers in their geometry. The ligands surrounding the Zn ions with alternate positions were not re-refined and the CMMS did flag the summation of ligand vectors. We conclude that Zn is incorporated from the crystallization conditions, and is the correct ion, but present in alternate positions causing the high *RSZD* for the OM. No further model building took place.

A structural model of fructokinase from *Brucella abortus* 2308 is given by PDB ID **4u7x.** The OM contains Na, which is present in the crystallization conditions. The XRFS data indicate Ni. PIXE data show Ni with a small amount of Zn. Of the four Na ions in the single chain of the OM, three have positive electron density surrounding them, one significantly so, and all have a high *B*-factor compared to the residues nearby. A later model for the same protein, PDB ID 4wjm, shows ethylene glycol in the place of one Na (supported by the electron density seen in **4u7x**), waters for two others, and nothing in place of the fourth. The Na ion with significant positive difference density was replaced with Ni, and the new model re-refined with PDB-REDO. This model gave an R and R_{free} of 0.195 and 0.229, and an *RSZD* of 2.5, compared to the original model 0.193 and 0.227 with an *RSZD* of 12.1. The Ni ion fits the electron density well with minimal difference density, and the difference density around the other Na ions was reduced. After re-refinement, the Na ions appear to be partially coordinated by water residues. The Ni *B*-factor is high, about 2-3× that of the coordinating residues and the water molecules, and it is probably present at less than unit occupancy. The difference electron density supports potential disorder around the Ni position, and the high *B*-factors may be due to this with the crystal consisting of bound and apo states. The occupancy was not refined. Given that *Brucella* strains require Fe, Mn, Zn, Mg and Ni[19], and the PIXE and XRFS data plus the crystallographic refinement, in this case the metal appears to be Ni.

There are four classes of β-lactamases: classes A–D. While classes A, C and D have an active site-serine, class B metallo-β--lactamases have a binuclear metal center in the active site that activates a water molecule. PDB ID **6ao1** is a structure of a class B β-lactamase from *Burkholderia phymatum*, a soil bacterium. Ca is modeled in the structure and was also present in the crystallization conditions. The XRFS data indicate Zn, Fe, Ni, Mn, and, Se with normalized ratios of 0.45, 0.44, 0.04, 0.04, and 0.03. Co is present at >2×LOD, and Cu at >1.5×LOD. The PIXE data are similar. There are four

chains in the OM, with a dual Ca ion site and a single Ca ion site in each chain. In chains A, B, and C, one Ca ion in the dual site has significant excess electron density and the lone one, negative electron density. Chain C has two extra Ca ions that appear well modeled in the electron density. In chain D, there is only a dual Ca ion site with one of the pair of Ca ions having excess electron density, and the other negative density. Metallo- β-lactamases require Zn in the binuclear metal site for activity[20] and the PIXE and XRFS data indicate that Zn and Fe are present at similar concentration. Metallo-β-lactamases can also use other metals, including Mn, Fe, and Cu, if the Zn concentration is low.[21] While trace amounts of Se are indicated, molecular replacement rather than Multiple-wavelength Anomalous Diffraction (MAD) phasing was used for the structure determination. Fe and Zn were modeled in the same ion positions in the dual metal site. The lone Ca ions were not changed but are likely present at less than unit occupancy. After refinement with PDB-REDO the *R* and R_{free} were 0.178 and 0.202 with an *RSZD* of 5.1 for Fe, 0.178 and 0.201 and an *RSZD* of -5.3 for Zn, and 0.178 and 0.203 with an *RSZD* of 10.8 for the OM. The difference electron density for both the Fe and Zn are comparable, with only minor differences present. Without anomalous structural data, it is not possible to accurately determine which ion, or mixture of ions are represented, but the XRFS data suggest an approximate 50/50 molar ratio of Fe and Zn. REFMAC5 was used to refine this mixture and the occupancy of the Ca ions. PDB-REDO was then rerun resulting in an *R* and R_{free} of 0.171 and 0.217, and *RSZD* of -2.1, and occupancies of ~50% but one site having a Fe and Zn occupancy of 74% and 26% respectively. In this case the new model has a reduced *R* but its R_{free} is slightly higher than for the OM. The *RSZD* is significantly lower, so the structure is now better modelled overall.

PDB ID **5unb** is a structural model of a putative deoxyribonuclease-2 from *Burkholderia thailandensis* in complex with copper.[22] The XRFS data did not show the presence of any metals at >3×LOD, although Ca and Ni had the highest LOD above any other, but were < 1. The PIXE data indicated Ca and Ni at a 0.65, 0.35 ratio, but Cu was present in the crystallization conditions. There are two Cu ions modeled in the first chain, both with reduced occupancy and displaying significant positive difference density associated with higher occupancy, and one Cu ion modeled in the second chain, also showing positive difference density. The OM had an *RSZD* -8.2 with an *R* and R_{free} of 0.148 and 0.180. The Cu sites are extensively described in this enzyme[22] but the experimental evidence provided by the X-ray structure does not support the presence of Cu at full occupancy, but at a higher occupancy than the OM. There is a water structure around the metal ions which does impact the difference density, but the Cu identification appears accurate and is present from the crystallization conditions.

PDB ID **3oce**, a model of fumarate lyase:delta crystallin from *Brucella melitensis* bound to Co, but Co was not detected in the XRFS or PIXE experiments. However, Co was also present in the crystallization conditions. The OM *RSZD* was - 4.0. The XRFS data indicated Zn, with the PIXE data agreeing and also showing the presence of Ni. It was noticeable that of the four chains, the modeled Co in two of them had shorter bond distances and were associated with negative density. These were modeled as Zn and Ni ions and the model re-refined. The *R* and R_{free} were 0.188 and 0.236 for Zn, and 0.193 and 0.239 for Ni, with *RSZD*s of 3.4 and 2.5, compared to the OM of 0.190 and 0.232. In this case, it seems likely that the metal sites contain a mixed occupancy of Zn and Co but without anomalous data, it is difficult to accurately define the relative metal contributions further.

As part of the study of potential tuberculosis drug targets[13], the structure of an Acyl-CoA thioesterase II TesB2 from *Mycobacterium marinum,* PDB ID **3u0a**, was determined. It was modeled with Na ions in both chains. No metals were detected in the PIXE or XRFS data. Na was present in the crystallization conditions. The three Na atoms, two in chain A and one in chain B, have *B*-factors significantly higher than surrounding atoms, and the negative density associated with the *RSZD* is from the second Na ion in the A chain, not seen in the other chain. In the absence of experimental evidence for the presence of Na, all these ions were replaced by water and the model re-refined. The resulting *R* and R_{free} were 0.183 and 0.232, the same as for the OM. The modeled waters displayed no difference density, with an *RSZD* of -0.9, compared to the original OM that had an *RSZD* of 3.8. While the Na could have come from the crystallization conditions, the RSZD would suggest that Na is not present.

PDB ID **6n1f** is a structural model of oxidoreductase, 2OG-Fe(II) oxygenase family, from *Burkholderia pseudomallei*. The XRFS data indicated Ni, and Zn in ratios of 0.89 and 0.11, the PIXE data no metal, and the OM showing Fe with one Fe ion in each of four chains each at 90% occupancy. The OM had *R* and R_{free} of 0.221 and 0.252 with an *RSZD* of -2.4 and replacing Fe with Ni gave *R* and R_{free} of 0.221 and 0.251. The experiential data support Ni, not Fe. The CMMS flagged Fe as an outlier. No further refinement took place.

PDB ID **3r4t** is a structural model of 4-aminobutyrate aminotransferase GabT from *Mycobacterium marinum*, a non-tuberculous mycobacterium that causes tuberculosis like illness in fish and can infect humans.[13] The structure was modeled as a single chain containing one Na ion. The XRFS data do not show the presence of any detectable metals at >2×LOD but Ca is the highest metal signal present at >1.0×LOD with the PIXE data also indicating Ca. The original *RSZD* was 1.0 and replacing the Na with Ca and re-refining did not improve the OM. The CMMS showed Na as acceptable to borderline, so while there is Ca in the protein

sample, the Na appears to be the correct metal, originating from the crystallization conditions.

SSGCID class II proteins – extra metals indicated experimentally

The metals present in the respective crystallization conditions were consistent with the models for PDB IDs **4gri**,[23] **3omf**,[24] **3r6f**, **3oca**, and **5j46** so no further re-refinement took place; the metals could be explained from those conditions.

PDB ID **4o5o** is a structural model of a 3-hydroxyacyl-CoA dehydrogenase from *Brucella suis*. In the OM there are two chains, one containing a fully occupied Zn ion with significant negative electron difference density and an *RSZD* of -24.3. The XRFS data show Ni with a small amount of Zn at >2×LOD. PIXE data also indicate Ni and Zn in a similar ratio. While the difference density could be from a lower occupancy of Zn, the data suggest that the Zn ion is misidentified and should be Ni. Re-refining with Ni modeled gave *R* and R_{free} of 0.155 and 0.178 compared to the 0.157 and 0.178 for the OM. At unit occupancy, the Ni ion displays significant negative difference density, and the metal site is presumably only partially occupied. Refining the occupancy with REFMAC5 gave 56% and this was used with PDB-REDO. A 50% occupancy reduces the R_{free} slightly to 0.176 and shows no difference density around the Ni site, with an *RSZD* of -1.7. The CMMS indicates that the modelled Zn ion is acceptable but has unoccupied sites in the coordination sphere. In this case, the experimental data suggest the metal should be Ni at lower than unit occupancy.

PDB ID **3uw2** is a structural model of a Phosphoglucomutase/phosphomannomutase family protein from *Burkholderia thailandensis*.[18] The OM has a single chain with a Zn ion displaying significant negative difference density. The XRFS data showed Zn, Fe, Ni, and Mn with ratios of 0.41, 0.24, 0.18, and 0.17 respectively. The PIXE data were similar. The three additional metals identified (Fe, Ni, and Mn) are all compatible with the difference electron density observed. The negative difference density indicates a lighter element, so the Zn was replaced with metals detected in the XRFS and PIXE measurements, and the model re-refined. The *R* and R_{free} for Fe were 0.180 and 0.214, for Ni were 0.180 and 0.215, and for Mn were 0.180 and 0.215, compared to the OM of 0.177 and 0.215. Fe, Ni, and Mn all displayed negative difference density. Zn, or any of these metals, could fit the experimental data, but at reduced occupancy. The occupancy of Zn was refined with the OM resulting in *R* and R_{free} of 0.168 and 0.222 for 67% occupancy, and an RSZD of -7.0 compared to the OM RSZD of -18.3. Phosphoglucomutase/phosphomannomutase requires divalent metal atoms as a cofactor and Mg is the most commonly associated metal.[25] This was modeled at the Zn position and the model re-refined. The resulting *R* and R_{free} were 0.176 and 0.213 and there was no difference density at the Mg ion site, an *RSZD* of -0.9. Mg, while undetectable with the XRFS and PIXE configurations used, is likely to be the correct metal.

PDB ID **4wok** is a structural model of UDP-glucose 4-epimerase from *Brucella ovis*, a pathogenic bacterium known to cause reproductive diseases in livestock. The protein is in complex with NAD. The OM is a single chain with a Zn ion surrounded by significant negative difference electron density. The XRFS data indicate K, Zn, Fe, Ca, and Ni at ratios of 0.78, 0.15, 0.05, 0.02, and 0.01. The PIXE data indicate K, Zn, Fe, and Ca. No metals were present in the crystallization conditions. The OM had an *R* and R_{free} of 0.167 and 0.193 and an *RSZD* of -15.1. Exploring Zn, indicated by the XRFS and PIXE data, the occupancy was refined with REFMAC5. Re-modeling with a reduced Zn occupancy of 65% gave an *R* and R_{free} of 0.161 and 0.196 with an RSZD of -1.8. Given the significant K signal, a K ion was modeled in the site resulting in an *R* and R_{free} of 0.177 and 0.205 respectively, and an *RSZD* of 3.3 with no noticeable difference density in the map. Based on the XRFS and PIXE data, and the refinement statistics, the correct metal in the model appears to be Zn at a reduced occupancy. There is evidence of K ions in other parts of the model, as indicated by the XRFS and PIXE data, but no further modeling or refinement took place.

PDB ID **4dz4** is a structural model of a hypothetical Agmatinase from *Burkholderia thailandensis*.[18] This is modeled as six chains with a bi-nuclear Mn ion site in each chain. Each site displays negative difference electron density. The XRFS data indicate Fe, Mn, and Ni with normalized ratios of 0.78, 0.19, and 0.03, with Se and Zn present at >2×LOD, supported by the PIXE data showing Fe, Mn, Ni and Zn, and Ca at lower signal levels. The OM failed the tortoize validation step with the PDB-REDO version used, and was refined with REFMAC5[3] separately. Replacing the Mn with Fe resulted in *R* and R_{free} values of 0.132 and 0.159 and displayed positive electron density enabling three missing residues to be added in one chain, and one in another. Refining this model further and lowering the Fe occupancy reduced the *R* and R_{free} to 0.131 and 0.159 respectively with an *RSZD* of 4.6 compared to the OM values of 0.156 and 0.180 and an *RSZD* of -13.7. The occupancy for the 12 Fe ions ranged from 70 to 80%. Given the XRFS and PIXE data showed Mn, another modeling step took place where Fe and Mn were modeled in the same site using the XRFS ratios. The resulting *R* and R_{free} were slightly improved, 0.131 and 0.158, with an *RSZD* of 3.1. Of the two sites in each chain, one favored Fe with the other having an Fe, Mn mixture. The CMMS showed Fe as mostly acceptable but flagged the percentage of the unoccupied sites in three cases out of 12. Chain A, C, and in particular F appeared to have positive difference electron density associated with a ligand. The CheckMyBlob server[26] was used to identify this, but the

electron density was too weak for an accurate identification. Two other regions of positive electron density were observed at the junction of three chains adjacent to Gln 293. Again, the density was too weak to model a ligand, but it could support sodium citrate which was present in the crystallization conditions. No further refinement was carried out. Given the XRFS and PIXE data, the metal ions are a mixture of predominately Fe and some Mn. Anomalous diffraction data would be needed to confirm this.

PDB ID **3r1j** is a structural model of alpha-ketoglutarate-dependent taurine dioxygenase from *Mycobacterium avium.*[18] It is modeled as two chains with an Fe ion in each chain coordinated to His 99, His 253 and Asp 101. The 2-His-1-carboxylate facial triad is found in a number of nonheme manganese, iron, and cobalt containing proteins.[27] Taurine dioxygenase has a strong binding affinity for Fe.[28] Both Fe ions have negative electron difference density with an *RSZD* of -11.9, and an R and R_{free} of 0.173 and 0.198. The XRFS data indicate Ni. PIXE data detected the presence of Ca, Ni and Fe, with ratios of 0.92, 0.05 and 0.02. Replacing the Fe with Ni and refining occupancy with REFMAC5 gave 55% and 61% occupancy in each chain. Re-refining resulted in R and R_{free} of 0.169 and 0.190 and an *RSZD* of 2.2. The CMMS indicates that Fe in the OM is border line to acceptable, as is Ni in the updated model. Based on the experimental data, the ions appear to be Ni.

PDB ID **4wjb** is a structural model of a putative amidohydrolase/peptidase from *Burkholderia cenocepacia*. It is modeled with four chains, one containing a Zn ion in two alternate positions with occupancies of 0.72 and 0.28, and excess negative difference electron density, a Zn ion in a second chain similar to the first, another in the third chain with slightly different occupancies of 0.80 and 0.20, and one in the fourth chain with occupancies of 0.66 and 0.34 but this time with excess positive difference electron density. The XRFS data show Zn, Fe, Ni, and Se, with normalized ratios of 0.79, 0.10, 0.08, and 0.04. The PIXE data indicate the presence of Zn, Fe, Ni and also Cu at > 2×LOD. The OM failed the tortoize validation step with the PDB-REDO version used, and was refined with REFMAC5[3] separately. It had R and R_{free} of 0.173 and 0.216 with an *RSZD* of -14.7. The CMMS considered the metal ions borderline in most of their parameters. Replacing the Zn ions with Fe gave an R and R_{free} of 0.172 and 0.216 with an *RSZD* of 4.2. Replacing the Zn ions with a mixture of Zn and Fe based on the XRFS data, approximately a 61%/39% mixture, resulted in R and R_{free} of 0.172 and 0.217, with an *RSZD* of 1.4. The CMMS result was borderline with improvement seen in the atomic contact criteria, but dubious ratings for the valence and nVESSUM criteria. Of note was that positive electron density appropriate for waters coordinated with the metal ions was visible, explaining those criteria values, but additional water molecules were not modeled. In this case it is difficult to assign metals due to the dual sites and the challenge in determining the occupancy of those sites, but the mixture appears to produce the best model statistics. No further refinement was carried out.

PDB ID **5umh** is a structural mode of catechol 1,2-dioxygenase protein from *Burkholderia multivorans*, a rare but emerging cause of bacterial meningitis.[29] There are two chains in the OM with a Zn ion and a Ca ion at occupancies of 0.84 and 0.86 respectively in one chain, and a Zn ion in the other chain with negative difference electron density. The XRFS data indicate Zn, Fe, and Ni at normalized ratios of 0.51, 0.40, and 0.08. The PIXE data are similar, with Fe, Zn, and Ni. It is likely that the difference density is due to the presence of a Zn ion at reduced occupancy, or that the ion is Fe. Catechol 1,2-dioxygenase is known to contain iron[30] and given these data, the Zn ions were replaced with Fe ions, and the new model re-refined. R and R_{free} for the new model were 0.132 and 0.164 with an *RSZD* of -4.9 compared to the OM of 0.138 and 0.169 with an *RSZD* of -10.8. The CMMS indicates that Zn is acceptable to borderline, however the correct metal in this case appears to be Fe or a combination of Fe and Zn.

PDB ID **4f0l** is the structural model of amidohydrolase from *Brucella melitensis*. The OM has two chains with an Fe ion in each. There is significant negative difference electron density at both the Fe ions with an *RSZD* of -10.4 and R and R_{free} *of 0.152 and 0.180.* The XRFS data indicate Fe, Ni, Mn, and Zn at ratios of 0.60, 0.20, 0.11, and 0.10. The PIXE data are mostly in agreement with a signal order of Zn, Fe, Ca and Mn and Ni at > 2×LOD. The negative difference density indicates partial occupancy or lower atomic weight ions. The Fe ions were refined at 50% occupancy and were also replaced with Ni and Mn. With 50% Fe occupancy, R and R_{free} were 0.141 and 0.194 with an *RSZD* of 3.6. The R and R_{free} for Ni were 0.136 and 0.183 with an *RSZD* of 1.4, and for Mn they were 0.142 and 0.176 with an *RSZD* of -3.4, compared with the OM value of -10.4. The CMMS indicates that Fe is acceptable to borderline with only an outlier flag for one of the ions' coordination sphere. Biochemically, studies on the ILL2 amidohydrolase hydrolase activity found that it required Mn or Co.[31] Refining the Fe occupancy specifically with REFMAC5 for the Fe case gave a R and R_{free} of 0.135 and 0.185 with an *RSZD* of -0.7 for 59% and 64% occupancy for the two Fe sites. However, based on the XRFS data, the refinement, and the RSZD, the Fe from the OM appears to be the correct metal. Given the biologically relevant Mn, and the Ni column used for purification, it is not surprising to see those metals, and it is possible that a small percentage of the molecules in the sample may incorporate them, but anomalous diffraction would be needed to explore this structurally.

PDB ID **3s6l** is a structural model of a YadA-like head domain of the trimeric autotransporter adhesin BoaA from

Burkholderia pseudomallei. The OM has five chains with four Zn ions in the first, two of which have positive difference density in the vicinity of the ions, and the other two have negative difference density. It has three Zn ions in the second chain, two with positive difference density in the vicinity of the ions, and one Zn ion in the third chain with negative difference density. There are three Zn ions in the fourth chain, all having adjacent positive difference density, and four in the fifth chain, again all having positive difference density. The XRFS data show Ni. The PIXE data also show Ni, Zn, and Cu. The OM also failed the tortoize validation step with the PDB-REDO version used and so was re-fined with REFMAC5[3] separately. For the OM, the R and R_{free} were 0.171 and 0.208 with an *RSZD* of 8.4. Replacing the Zn ions with Ni and re-refining resulted in R and R_{free} of 0.170 and 0.209 with an *RSZD* of 2.5. There appeared to be evidence of a ligand interacting with one of the Ni ions that could not be explained based on the experimental conditions. Testing with CheckMyBlob suggested a 53% probability of a rare ligand, not currently sampled in the PDB. The metal appears to be Ni with an unidentified ligand present. No further refinement was carried out.

PDB ID **5u8o** is a structural model of beta-lactamase domain protein from *Burkholderia multivorans*. The OM has four chains with a dual metal site with Zn ions in each chain and a single Ca ion in one chain. Three of the chains display varying degrees of negative difference electron density. The XRFS data indicate Fe, Zn, Mn, Se, and Ni with normalized ratios of 0.61, 0.21, 0.08, 0.06, and 0.05 respectively. The PIXE data are comparable. The Zn ions were replaced in turn with Fe and Mn and the model re-refined. The Fe model gave R and R_{free} of 0.178 and 0.219, Mn 0.178 and 0.219, with the OM giving 0.178 and 0.219. The *RSZD* for the OM was -9.1 for the Zn, and -3.6 and -3.2, for Fe and Mn respectively. Given that Zn was present in the crystallization conditions, it is difficult to assign the metals based on the available information but it seems likely that they are all Fe since these give the highest experimental signal.

PDB ID **4di0** is a structural model of rubrerythrin from *Burkholderia pseudomallei*. The OM has two chains with one Fe ion in the first, and two in the second. The XRFS data indicate Fe, Zn, Mn, Ni, Ca, and Co with normalized ratios of 0.61. 0.24, 0.09, 0.03, 0.01, and 0.01. The PIXE data show Fe, Mn, Zn, and Ca, and also Ni at > 2×LOD. The difference electron density around the Fe ions is negative indicating partial occupancy or a lower atomic weight element. The original model had an R and R_{free} of 0.170 and 0.199 and an RSZD of -9.1 with some unreasonable *B*-factors for the chain. This was corrected and the occupancies of the Fe ions were refined with REFMAC5 from 62% to 75%, yielding an R and R_{free} of 0.149 and 0.191 and an *RSZD* of -4.3. The CMMS indicates that Fe in the OM is borderline to acceptable with unoccupied sites in the coordination sphere for two of the Fe ions. Both the XRFS and PIXE data show multiple metals at significant ratios. It is likely that the correct metal is Fe with other metals present in a mixture. The full details would have to be found using anomalous techniques.

PDB ID **5kob** is a structural model of a peptide deformylase from *Burkholderia xenovorans*. Peptide deformylase is essential in a variety of pathogenic bacteria but is not required for cytoplasmic protein synthesis in eukaryotes and is therefore an interesting potential target for antibacterial agents. Peptide deformylase behaves as a metalloenzyme, co-purifying with Zn[32] and Ni[33]. The OM has four chains, each containing an Fe ion, three of which have mixed difference density associated with them. All the Fe ions are modeled at partial occupancies of 0.88. The R and R_{free} were 0.165 and 0.192 with an *RSZD* of -6.0. It is notable that a small part of one chain had 12 residues with low *B*-factors, leading to positive difference density along chains. The XRFS data show Zn, Fe, and Ni at a normalized ratio of 0.55, 0.34, and 0.11. The PIXE data indicate Fe. The abnormal *B*-factors were set to 30 Å^2, and guided by the XRFS data results, Fe was replaced with Zn and the model re-refined with a reduced Zn occupancy of 65-75%. This resulted in an R and R_{free} of 0.156 and 0.192, and a *RSZD* of 3.3, and also revealed formic acid close to the metal sites from the crystallization conditions. It is likely that the partial occupancy Zn sites, 65-75%, are a mixture of Zn, Fe, and possibly Ni, but anomalous diffraction would be needed to confirm this. The model was not refined further.

PDB ID **6n7l** is a structural model of an alcohol dehydrogenase from *Elizabethkingia anophelis* NUHP1. It has eight chains with a Zn ion in each. The XRFS data indicate Zn and Ni in a ratio of 0.96 and 0.04 respectively. The PIXE data are similar. The OM had refined well on a previous version of PDB-REDO with R and R_{free} of 0.154 and 0.188 and *RSZD* of -6.2 but failed the tortoize validation step with the newer version used. The CMMS indicated that Zn was mostly acceptable but with some borderline flags in *B*-factor, valence and nVECSU. It was refined with REFMAC5[3] separately and gave R and R_{free} of 0.148 and 0.188 and an *RSZD* of -3.8. The metal appears to be Zn and no further refinement was carried out.

PDB ID **6d6j** is a structural model of an HIT family hydrolase from *Legionella pneumophila Philadelphia 1*. Two chains are modeled with Zn ions, both at occupancies close to 1. The XRFS data indicate Zn, Ni, and Se, with a normalized signal of 0.97, 0.02, and 0.01, respectively. The PIXE data are similar but also show a small amount of Fe and Co. There is some positive difference density, and an *RSZD* of 5.3, presumably associated with a slight reduction in occupancy. No re-refinement took place.

PDB ID **3md7** is a class B β-beta-lactamase-like protein from *Brucella melitensis* bound to guanosine-5'-monophosphate

and was modeled with Mn in the binuclear metal site.[34] There are three Mn atoms modeled, two in the binuclear site and one elsewhere. One of the Mn atoms in the binuclear site is modeled at 0.75 occupancy and displays excess electron density, an *RSZD* of 5.1, and indications that surrounding residues may not be optimally orientated. There are also K and Na ions present in the model. The XRFS data indicate Fe, Mn, Zn, Se, and Ni, at ratios of 0.43, 0.25, 0.17, 0.09, and 0.05 respectively. Ca was present at >2×LOD. The PIXE data were similar but also showed the presence of K at >2×LOD. In determining the model for this structure, anomalous signals for Mn, Zn, K and Ni were compared at the three different X-ray wavelengths used for the structure determination, and Mn was deemed to give the best fit to the signals.[34] From that study, Fe was also consistent with the observed anomalous scattering. The model was not re-refined due to the confirmation from the original study that the most likely metal was Mn.

PDB ID **4j5i**, is a structural model of an alpha-ketoglutarate-dependent taurine dioxygenase from *Mycobacterium smegmatis*.[13] It was modeled as eight chains each containing an Fe ion and one chain also containing a Mg ion. The XRFS data indicates Ni, Zn, and Fe, with normalized signals of 0.81, 0.11, and 0.08 respectively, and Cu at > 2×LOD. The PIXE data agree, showing Ni and Fe and also Zn at > 2×LOD. The crystallization conditions contained Mg, Li, and Na. The model reports a ligand with the formula $C_4H_9NO_2$ but there is an issue with this assignment. The separate components of the moiety were replaced with alpha-aminobutyric acid which fits the electron density well and the OM was re-refined. Fe then was replaced with Ni and the model again re-refined. R and R_{free} for the Ni model were 0.197 and 0.230, the same as the OM, but now supported by the experimental evidence for the Ni metal present, rather than being Fe. The new *RSZD* was 2.1 compared with the original value of 4.6. It is likely that the Ni has been opportunistically incorporated at the sample purification step where a Ni-affinity process is part of the pipeline.[35]

PDB ID **5umf** is a structural model of a ribulose-phosphate 3-epimerase from *Neisseria gonorrhoeae*. The OM has three chains, each with a Zn ion. While there is no difference density around the Zn ions, in each case there appears to be positive density in the form of a possible ligand in the vicinity of the ions. The XRFS data indicate Zn and Ni at > 2×LOD. The PIXE data detected Zn and Fe with Ni also present at > 2×LOD. In the OM, the Zn ions are modeled at reduced occupancy ranging from 0.57 to 0.60. Replacing the Zn with Fe and Mg results in R and R_{free} of 0.125 and 0.149 for Fe, and 0.125 and 0.150 for Mg following re-refinement, compared to the original 0.130 and 0.151 with an *RSZD* of -2.5. Modeling D-ribulose as the ligand with 50% occupancy reduced the R and R_{free} to 0.122 and 0.148 but raised the *RSZD* slightly to -2.7, indicating a reduced occupancy of Zn or the presence of a mixture of Zn and Ni. No further refinement took place.

PDB ID **3o0m** is a structural model of a Zn-bound histidine triad family protein from *Mycobacterium smegmatis*.[13] There are two chains each having a single Zn ion in each. The XRFS data indicate Zn, and Cu with ratios of 0.98, and 0.02, with Ni present at > 1.5×LOD, and the PIXE data show Zn and Ni. There is minimal difference density at the Zn ion sites and given the ratio of Ni and Zn signals (0.96 and 0.04), no reason is suggested for replacing the Zn ions with Ni. No re-refinement took place.

PDB ID **6caz** is peptide deformylase from *Legionella pneumophila*, a gram-negative facultative intracellular human pathogen that can cause fatal Legionnaires' disease. The OM is a single chain with one Ni ion coordinated to three residues and a Cl atom. The XRFS data indicate Zn, and Ni, with normalized ratios of 0.93, and 0.07 respectively, with Se present at >2.0×LOD. The PIXE data indicate Zn and Ca, with Ni also present at >2×LOD. The single Ni site displays mixed difference electron density but Ni is known to bind to deformylase.[33] Given the experimental data, Zn was modeled into the Ni site, and the model refined. R and R_{free} for the Zn ions were 0.173 and 0.198 with an *RSZD* of -3.1 compared to 0.189 and 0.214 and an *RSZD* of -4.9 for the OM. The CMMS flags Ni in the OM as acceptable but the geometry as an outlier. Zn is flagged as acceptable in all categories. We conclude from the experimental data that the correct metal is Zn.

References

(1) Schmier, B. J.; Seetharaman, J.; Deutscher, M. P.; Hunt, J. F.; Malhotra, A. The structure and enzymatic properties of a novel RNase II family enzyme from Deinococcus radiodurans. *J Mol Biol* **2012**, *415* (3), 547-559. DOI: 10.1016/j.jmb.2011.11.031 From NLM Medline.

(2) Forouhar, F.; Hussain, M.; Farid, R.; Benach, J.; Abashidze, M.; Edstrom, W. C.; Vorobiev, S. M.; Xiao, R.; Acton, T. B.; Fu, Z.; et al. Crystal structures of two bacterial 3-hydroxy-3-methylglutaryl-CoA lyases suggest a common catalytic mechanism among a family of TIM barrel metalloenzymes cleaving carbon-carbon bonds. *J Biol Chem* **2006**, *281* (11), 7533-7545. DOI: 10.1074/jbc.M507996200 From NLM Medline.

(3) Murshudov, G. N.; Skubak, P.; Lebedev, A. A.; Pannu, N. S.; Steiner, R. A.; Nicholls, R. A.; Winn, M. D.; Long, F.; Vagin, A. A. REFMAC5 for the refinement of macromolecular crystal structures. *Acta Crystallogr D Biol Crystallogr* **2011**, *67* (Pt 4), 355-367. DOI: 10.1107/S0907444911001314 From NLM Medline.

(4) Feng, H.; Dong, L.; Cao, W. Catalytic Mechanism of Endonuclease V: A Catalytic and Regulatory Two-Metal Model. *Biochemistry* **2006**, *45* (34), 10251-10259. DOI: 10.1021/bi060512b.

(5) Aimin, L.; Tingfeng, L.; Rong, F. Amidohydrolase Superfamily. In *eLS*.

(6) Gueroult, M.; Picot, D.; Abi-Ghanem, J.; Hartmann, B.; Baaden, M. How cations can assist DNase I in DNA binding and

hydrolysis. *Plos Comput Biol* **2010**, *6* (11), e1001000. DOI: 10.1371/journal.pcbi.1001000 From NLM Medline.

(7) Arbing, M. A.; Handelman, S. K.; Kuzin, A. P.; Verdon, G.; Wang, C.; Su, M.; Rothenbacher, F. P.; Abashidze, M.; Liu, M.; Hurley, J. M.; et al. Crystal structures of Phd-Doc, HigA, and YeeU establish multiple evolutionary links between microbial growth-regulating toxin-antitoxin systems. *Structure* **2010**, *18* (8), 996-1010. DOI: 10.1016/j.str.2010.04.018 From NLM Medline.

(8) Masuda, H.; Tan, Q.; Awano, N.; Wu, K. P.; Inouye, M. YeeU enhances the bundling of cytoskeletal polymers of MreB and FtsZ, antagonizing the CbtA (YeeV) toxicity in Escherichia coli. *Mol Microbiol* **2012**, *84* (5), 979-989. DOI: 10.1111/j.1365-2958.2012.08068.x From NLM Medline.

(9) Abbott, D. W.; Gilbert, H. J.; Boraston, A. B. The active site of oligogalacturonate lyase provides unique insights into cytoplasmic oligogalacturonate beta-elimination. *J Biol Chem* **2010**, *285* (50), 39029-39038. DOI: 10.1074/jbc.M110.153981 From NLM Medline.

(10) Sreedhar, R.; Kaul Tiku, P. Cupincin: A Unique Protease Purified from Rice (Oryza sativa L.) Bran Is a New Member of the Cupin Superfamily. *PLoS One* **2016**, *11* (4), e0152819. DOI: 10.1371/journal.pone.0152819 From NLM Medline.

(11) McCoy, A. J.; Grosse-Kunstleve, R. W.; Adams, P. D.; Winn, M. D.; Storoni, L. C.; Read, R. J. Phaser crystallographic software. *J Appl Crystallogr* **2007**, *40* (Pt 4), 658-674. DOI: 10.1107/S0021889807021206 From NLM PubMed-not-MEDLINE.

(12) Patterson, E. I.; Nanson, J. D.; Abendroth, J.; Bryan, C.; Sankaran, B.; Myler, P. J.; Forwood, J. K. Structural characterization of beta-ketoacyl ACP synthase I bound to platencin and fragment screening molecules at two substrate binding sites. *Proteins* **2020**, *88* (1), 47-56. DOI: 10.1002/prot.25765 From NLM Medline.

(13) Baugh, L.; Phan, I.; Begley, D. W.; Clifton, M. C.; Armour, B.; Dranow, D. M.; Taylor, B. M.; Muruthi, M. M.; Abendroth, J.; Fairman, J. W.; et al. Increasing the structural coverage of tuberculosis drug targets. *Tuberculosis (Edinb)* **2015**, *95* (2), 142-148. DOI: 10.1016/j.tube.2014.12.003 From NLM Medline.

(14) Varela-Ramirez, A.; Abendroth, J.; Mejia, A. A.; Phan, I. Q.; Lorimer, D. D.; Edwards, T. E.; Aguilera, R. J. Structure of acid deoxyribonuclease. *Nucleic Acids Res* **2017**, *45* (10), 6217-6227. DOI: 10.1093/nar/gkx222 From NLM Medline.

(15) Kerr, K. G.; Snelling, A. M. Pseudomonas aeruginosa: a formidable and ever-present adversary. *Journal of Hospital Infection* **2009**, *73* (4), 338-344. DOI: 10.1016/j.jhin.2009.04.020.

(16) Rohdich, F.; Eisenreich, W.; Wungsintaweekul, J.; Hecht, S.; Schuhr, C. A.; Bacher, A. Biosynthesis of terpenoids. 2C-Methyl-D-erythritol 2,4-cyclodiphosphate synthase (IspF) from Plasmodium falciparum. *Eur J Biochem* **2001**, *268* (11), 3190-3197. DOI: 10.1046/j.1432-1327.2001.02204.x From NLM Medline.

(17) Kemp, L. E.; Bond, C. S.; Hunter, W. N. Structure of 2C-methyl-D-erythritol 2,4- cyclodiphosphate synthase: an essential enzyme for isoprenoid biosynthesis and target for antimicrobial drug development. *Proc Natl Acad Sci U S A* **2002**, *99* (10), 6591-6596. DOI: 10.1073/pnas.102679799 From NLM Medline.

(18) Baugh, L.; Gallagher, L. A.; Patrapuvich, R.; Clifton, M. C.; Gardberg, A. S.; Edwards, T. E.; Armour, B.; Begley, D. W.; Dieterich, S. H.; Dranow, D. M.; et al. Combining functional and structural genomics to sample the essential Burkholderia structome. *PLoS One* **2013**, *8* (1), e53851. DOI: 10.1371/journal.pone.0053851 From NLM Medline.

(19) Roop, R. M., 2nd. Metal acquisition and virulence in Brucella. *Anim Health Res Rev* **2012**, *13* (1), 10-20. DOI: 10.1017/S1466252312000047 From NLM Medline.

(20) Bahr, G.; Gonzalez, L. J.; Vila, A. J. Metallo-beta-lactamases in the Age of Multidrug Resistance: From Structure and Mechanism to Evolution, Dissemination, and Inhibitor Design. *Chem Rev* **2021**, *121* (13), 7957-8094. DOI: 10.1021/acs.chemrev.1c00138 From NLM Medline.

(21) Cahill, S. T.; Tarhonskaya, H.; Rydzik, A. M.; Flashman, E.; McDonough, M. A.; Schofield, C. J.; Brem, J. Use of ferrous iron by metallo-beta-lactamases. *J Inorg Biochem* **2016**, *163*, 185-193. DOI: 10.1016/j.jinorgbio.2016.07.013 From NLM Medline.

(22) Varela-Ramirez, A.; Abendroth, J.; Mejia, A. A.; Phan, I. Q.; Lorimer, D. D.; Edwards, T. E.; Aguilera, R. J. Structure of acid deoxyribonuclease. *Nucleic Acids Res* **2017**, *45* (10), 6217-6227. DOI: 10.1093/nar/gkx222 (acccessed 9/19/2024).

(23) Moen, S. O.; Edwards, T. E.; Dranow, D. M.; Clifton, M. C.; Sankaran, B.; Van Voorhis, W. C.; Sharma, A.; Manoil, C.; Staker, B. L.; Myler, P. J.; et al. Ligand co-crystallization of aminoacyl-tRNA synthetases from infectious disease organisms. *Sci Rep* **2017**, *7* (1), 223. DOI: 10.1038/s41598-017-00367-6 From NLM Medline.

(24) Lorimer, D. D.; Choi, R.; Abramov, A.; Nakazawa Hewitt, S.; Gardberg, A. S.; Van Voorhis, W. C.; Staker, B. L.; Myler, P. J.; Edwards, T. E. Structures of a histidine triad family protein from Entamoeba histolytica bound to sulfate, AMP and GMP. *Acta crystallographica. Section F, Structural biology communications* **2015**, *71* (Pt 5), 572-576. DOI: 10.1107/S2053230X1500237X From NLM Medline.

(25) Stiers, K. M.; Muenks, A. G.; Beamer, L. J. Chapter Eight - Biology, Mechanism, and Structure of Enzymes in the α-d-Phosphohexomutase Superfamily. In *Advances in Protein Chemistry and Structural Biology*, Karabencheva-Christova, T. Ed.; Vol. 109; Academic Press, 2017; pp 265-304.

(26) Kowiel, M.; Brzezinski, D.; Porebski, P. J.; Shabalin, I. G.; Jaskolski, M.; Minor, W. Automatic recognition of ligands in electron density by machine learning. *Bioinformatics* **2019**, *35* (3), 452-461. DOI: 10.1093/bioinformatics/bty626 From NLM Medline.

(27) Li, M. The Thermodynamics of Divalent Metals Binding to Taurine/α-Ketoglutarate-Dependent Oxygenase (TAUD). *Biophys J* **2017**, *112* (3), 347a-348a. DOI: 10.1016/j.bpj.2016.11.1886 (acccessed 2024/09/19).

(28) Grzyska, P. K.; Hausinger, R. P.; Proshlyakov, D. A. Metal and substrate binding to an Fe(II) dioxygenase resolved by UV spectroscopy with global regression analysis. *Analytical Biochemistry* **2010**, *399* (1), 64-71. DOI: https://doi.org/10.1016/j.ab.2009.11.022.

(29) Peralta, D. P.; Chang, A. Y.; Ariza-Hutchinson, A.; Ho, C. A. Burkholderia multivorans: A rare yet emerging cause of bacterial meningitis. *IDCases* **2018**, *11*, 61-63. DOI: https://doi.org/10.1016/j.idcr.2018.01.002.

(30) Di Nardo, G.; Roggero, C.; Campolongo, S.; Valetti, F.; Trotta, F.; Gilardi, G. Catalytic properties of catechol 1,2-dioxygenase from Acinetobacter radioresistens S13 immobilized on nanosponges. *Dalton Transactions* **2009**, (33), 6507-6512, 10.1039/B903105G. DOI: 10.1039/B903105G.

(31) Rampey, R. A.; Woodward, A. W.; Hobbs, B. N.; Tierney, M. P.; Lahner, B.; Salt, D. E.; Bartel, B. An Arabidopsis Basic Helix-Loop-Helix Leucine Zipper Protein Modulates Metal Homeostasis and Auxin Conjugate Responsiveness. *Genetics* **2006**, *174* (4), 1841-1857. DOI: 10.1534/genetics.106.061044 (acccessed 9/20/2024).

(32) Meinnel, T.; Lazennec, C.; Blanquet, S. Mapping of the active site zinc ligands of peptide deformylase. *J Mol Biol* **1995**, *254* (2), 175-183. DOI: 10.1006/jmbi.1995.0609 From NLM Medline.

(33) Ragusa, S.; Blanquet, S.; Meinnel, T. Control of peptide deformylase activity by metal cations. *J Mol Biol* **1998**, *280* (3), 515-523. DOI: 10.1006/jmbi.1998.1883 From NLM Medline.

(34) Abendroth, J.; Sankaran, B.; Edwards, T. E.; Gardberg, A. S.; Dieterich, S.; Bhandari, J.; Napuli, A. J.; Van Voorhis, W. C.; Staker, B. L.; Myler, P. J.; et al. BrabA.11339.a: anomalous diffraction and ligand binding guide towards the elucidation of the function of a 'putative beta-lactamase-like protein' from Brucella melitensis. *Acta Crystallogr Sect F Structural biology communications* **2011**, *67* (Pt 9), 1106-1112. DOI: 10.1107/S1744309111010220 From NLM Medline.

(35) Xiao, R.; Anderson, S.; Aramini, J.; Belote, R.; Buchwald, W. A.; Ciccosanti, C.; Conover, K.; Everett, J. K.; Hamilton, K.; Huang, Y. J.; et al. The high-throughput protein sample production platform of the Northeast Structural Genomics Consortium. *J Struct Biol* **2010**, *172* (1), 21-33. DOI: S1047-8477(10)00225-X [pii] 10.1016/j.jsb.2010.07.011.
